# GWAS reveal SUBER GENE1-mediated suberization via type one phosphatases

**DOI:** 10.1038/s41477-026-02292-x

**Published:** 2026-05-05

**Authors:** Jian-Pu Han, Linnka Lefebvre-Legendre, Jun Yu, Maria Beatriz Capitão, Chloé Beaulieu, Kay Gully, Vinay Shukla, Yibo Wu, Andreas Boland, Christiane Nawrath, Marie Barberon

**Affiliations:** 1 Department of Plant Sciences, University of Geneva, Geneva, Switzerland; 2 Department of Molecular and Cellular Biology, University of Geneva, Geneva, Switzerland; 3 MARBEC, Univ Montpellier, CNRS, Ifremer, IRD, Sète, France; 4 Department of Plant Molecular Biology, University of Lausanne, Lausanne, Switzerland; 5 Chemical Biology Mass Spectrometry Platform, Faculty of Sciences, University of Geneva, Geneva, Switzerland; 6 Present Address: School of Biosciences, University of Nottingham, Sutton Bonington, UK; 7 Present Address: Department of Biomedical Sciences, University of Lausanne, Lausanne, Switzerland

**Keywords:** Patterning, Natural variation in plants

## Abstract

Suberin deposition in the root endodermis is critical for plant nutrient acquisition and environmental adaptation. Here we used an unbiased forward genetic approach based on natural variation across 284 *Arabidopsis thaliana* accessions to identify novel regulators of endodermal suberization. This screen revealed striking diversity in suberin levels and patterns, uncovering broader roles for suberin beyond those observed in the reference accession Col-0. A genome-wide association study pinpointed *SUBER GENE1* (*SBG1*), a previously uncharacterized gene encoding a 129-amino-acid protein, as a key regulator of suberin deposition. SBG1 acts through physical interaction with type one protein phosphatases (TOPPs) via conserved SILK and RVxF motifs. Disrupting this interaction abolishes SBG1 function, while *topp* mutants exhibit enhanced endodermal suberization, mirroring *SBG1* overexpression. Our findings uncover a previously unknown regulatory module linking suberin formation to TOPP activity and abscisic acid signalling, and provide a framework for improving plant stress resilience through targeted manipulation of root barrier properties.

## Abstract (web summary)

A genetic screen across *Arabidopsis* accessions identifies SBG1 as a regulator of root abscisic acid response and endodermal suberin deposition via type 1 protein phosphatases, revealing how natural variation shapes root barriers and stress responses.

## Main

**Fig. 1:**
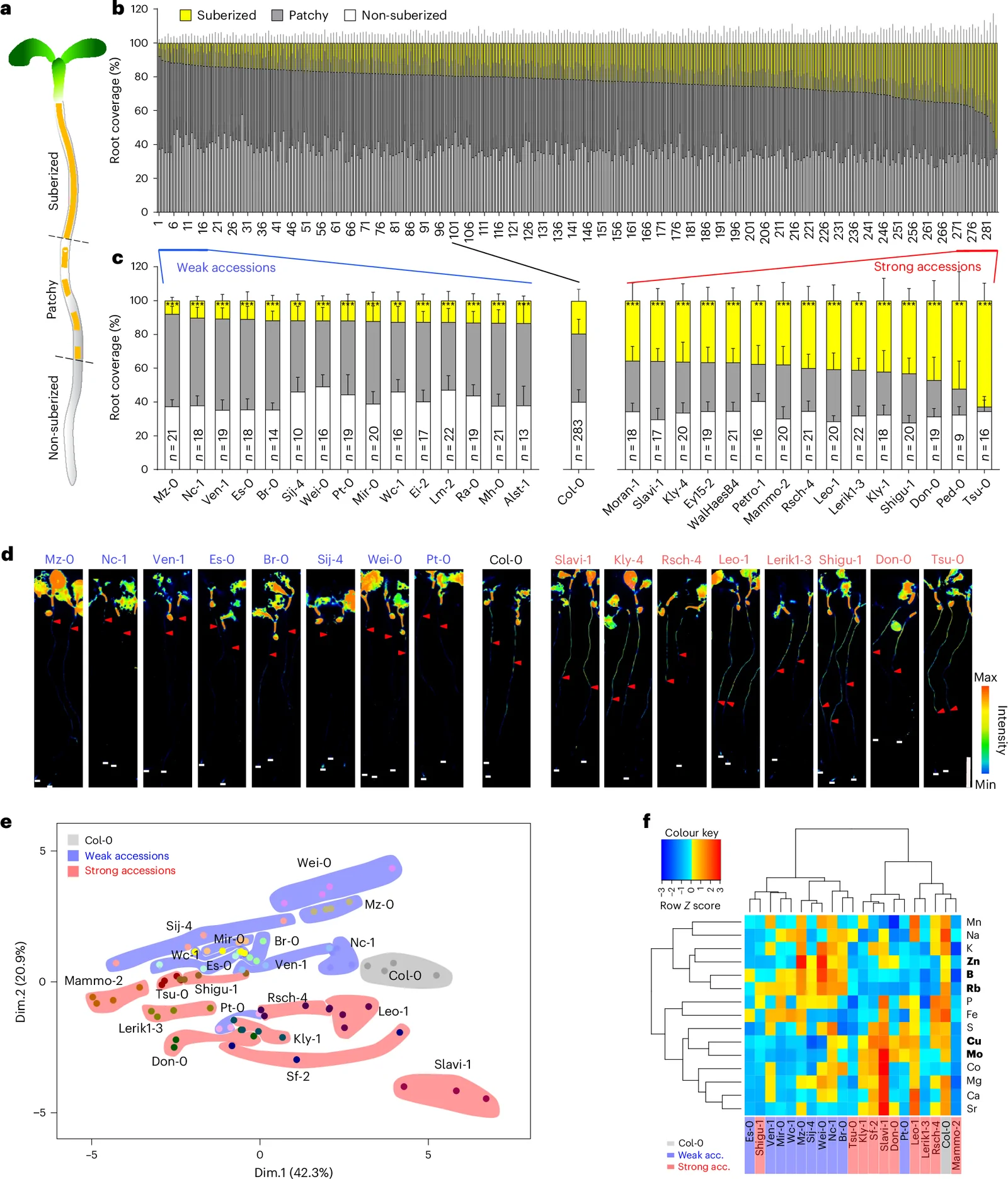
Natural variation in suberin deposition among *Arabidopsis* accessions. **a**, Schematic view of the three zones of suberization in roots. **b**, Quantification of suberin deposition in 4-day-old roots of 284 *Arabidopsis* accessions using fluorol yellow staining. Accessions are sorted by the percentage of suberized zone. Data are presented as stacked columns showing the mean percentage of each suberization zone ±s.d., with exact *n* for each accession provided in Supplementary Table 1 alongside numeric values. **c**, Comparison of suberin deposition in Col-0 and selected accessions representing the extremes (weak and strong) of the distribution shown in **b**). Statistical differences in the suberized zone were assessed by one-way analysis of variance (ANOVA) followed by Dunnett’s test and are presented for the comparison to Col-0 (**P < 0.01, ***P < 0.001, with exact *P* values provided in Source Data Fig. 1 and *n* shown on the graph). **d**, Whole-mount images of fluorol yellow-stained roots from selected extreme accessions. Red arrowheads indicate the onset of the suberized zone; white lines mark the root tip. Scale bar, 2 mm. Experiment was repeated 3 times independently with similar results. **e**, PCA of ionomic profiles in Col-0 and 20 extreme accessions. Accessions with weak and strong suberization are indicated with blue and red areas, respectively (hand drawn). *n* = 4 except for Es-0, Pt-0, Sf-2, Slavi-1 and Ven-1 where *n* = 3. **f**, Hierarchical clustering of ionomic profiles from the same set of extreme accessions. The 5 elements contributing most to the clustering are highlighted in bold. Data for **e** and **f** are available in Supplementary Table 3. Source data

Plant roots serve as the primary interface between plants and the soil, playing a critical role in nutrient acquisition and water uptake. To regulate the movement of water, ions and nutrients, roots have evolved complex anatomical structures, including the deposition of suberin in the root endodermis. Suberin, a hydrophobic biopolymer, forms a selective barrier that not only controls radial transport but also protects plants from environmental stresses such as drought, salinity, nutrient imbalances and pathogens^1^. The pattern of suberin deposition in the root endodermis is precisely regulated during root development^1^. In *Arabidopsis thaliana*, endodermal differentiation progresses through distinct stages. Initially, Casparian strips (CS) form an apoplastic barrier (non-suberized region) in the endodermis. Following this, patches of endodermal cells undergo suberization (patchy suberization zone), where suberin is selectively deposited. Eventually, the entire endodermal layer becomes uniformly suberized (suberized zone) (Fig. 1a). At the molecular level, the pattern of endodermal suberization is a highly regulated process, orchestrated by the interplay of several hormones and signalling pathways^1^. Among them, abscisic acid (ABA) plays a central role in both developmental and stress-induced suberin formation^2^. Exogenous ABA treatments strongly upregulate the expression of genes involved in suberin biosynthesis, monomer transport and polymerization, and lead to ectopic endodermal suberization close to the root tip^2–7^. Conversely, ABA-insensitive mutants, including lines expressing the dominant-negative *abi1-1* allele of *ABA INSENSITIVE1* specifically in the endodermis, exhibit markedly reduced suberin deposition^2,3,8,9^. These findings demonstrate that endodermal ABA signalling is not only essential for stress-induced suberization but also required for proper developmental establishment of the endodermal suberin barrier. In addition, CASPARIAN STRIP INTEGRITY FACTOR (CIF) peptides, interacting with the Leucine-Rich Repeat Receptor-Like Kinase SGN3/GSO1 (SCHENGEN3/GASSHO1), regulate the integrity and proper function of CS by reinforcing endodermal barriers, including suberization^10,11^, independently of ABA^3,8^. Recent studies have also highlighted MYELOBLASTOSIS (MYB) transcription factors, such as MYB9, MYB39, MYB41, MYB53, MYB92 and others^3,7,12,13^, acting downstream of ABA, as key regulators controlling suberin biosynthesis, transport and polymerization. The complexity of this regulatory network underscores suberin’s central role in stress adaptation and environmental acclimation. Despite substantial progress in identifying the molecular components involved in suberin biosynthesis and regulation, no unbiased forward genetic screen has yet been conducted to discover regulators of endodermal suberization. Furthermore, natural variation in suberin deposition across plant species and genotypes remains largely unexplored.

In this study, we investigated natural variation in suberin deposition within the root endodermis. By analysing a set of 284 *A. thaliana* accessions, we identified natural variants with distinct suberin deposition patterns and demonstrated that suberin plays a central role in mineral accumulation, extending beyond its previously established functions in the reference ecotype Col-0. Through a genome-wide association study (GWAS), we identified *SUBER GENE1* (*SBG1*), a previously uncharacterized gene encoding a 129-amino-acid protein that plays a key role in suberin regulation. We further discovered that SBG1 interacts with type one protein phosphatases (TOPPs) via conserved SILK and RVxF motifs and inhibits the phosphatase activity, positioning it as a novel TOPPs regulator involved in fine tuning endodermal suberization. These findings reveal a previously unknown regulatory mechanism governing suberin formation and provide new insights into the genetic determinants of suberization and its role in plant adaptation to environmental conditions.

## Results

### Natural variation for endodermal suberin

**Extended Data Fig. 1:**
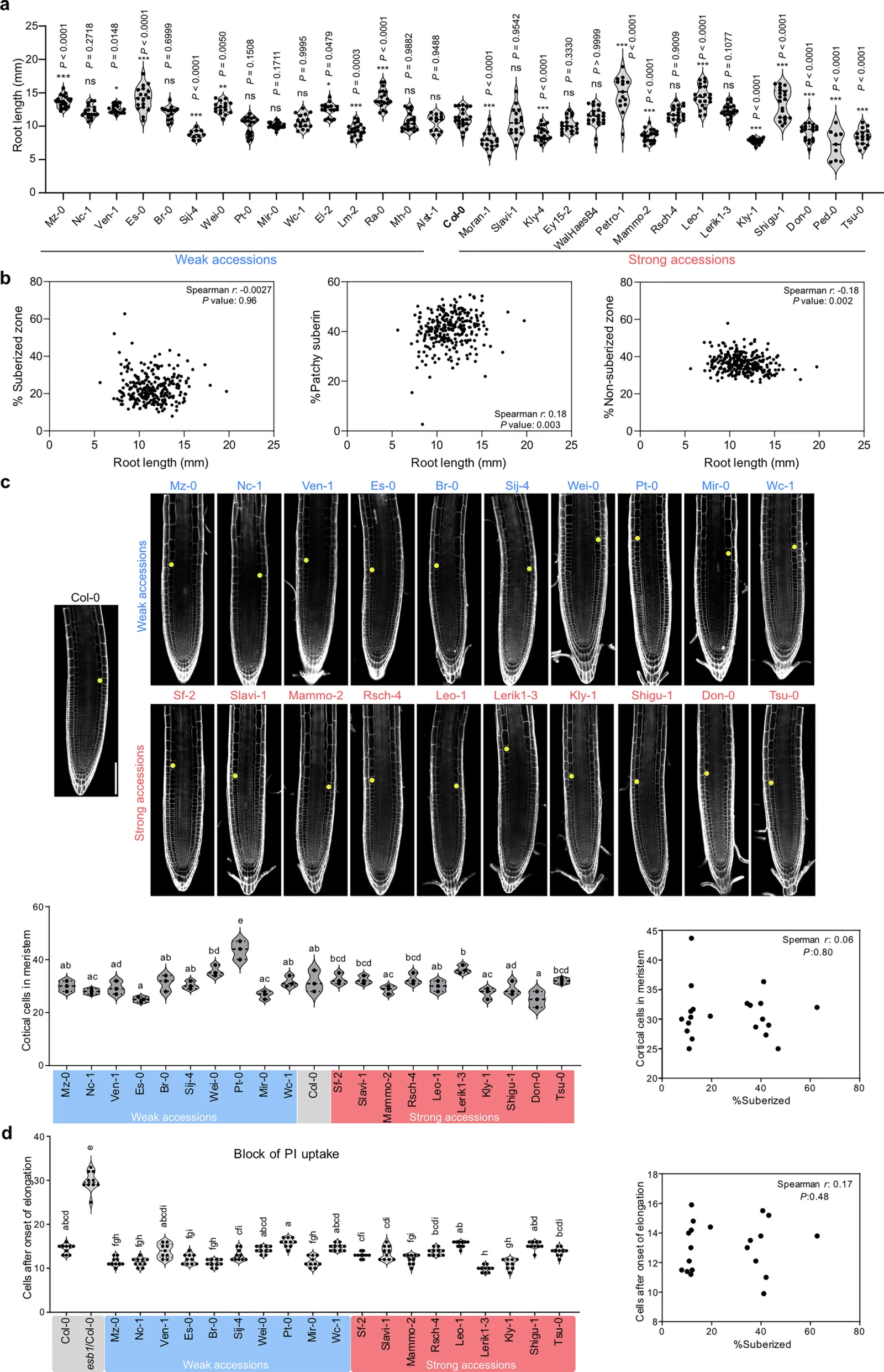
Root morphology of extreme accessions. **a**, Primary root length of 4-day-old Col-0 and 30 extreme accessions selected from Fig. 1a. Data are presented as violin plots with individual data points overlaid, with exact *n* for each accession provided in Supplementary Table 1 alongside numeric values *n*. Thick dashed lines in the violin plots indicate the median, while dotted lines represent the first and third quartiles. Statistical differences were assessed by one-way ANOVA followed by Dunnett’s test and are presented for the comparison to Col-0 (*P < 0.05, **P < 0.01, ***P < 0.001). **b**, Two-tailed Spearman correlation analysis between primary root length and the extent of fully suberized, patchy suberized, or non-suberized zones across the 284 accessions. Each dot represents the mean value for one accession. **c**, Root morphology in 5-day-old extreme accessions (upper panels). Cell walls were stained with PI (grey). Yellow dots mark the onset of cortex cell elongation. Scale bar, 100 µm. Violin plots presented as in (**a**) showing the number of cortical cells under division in the meristem, *n* = 3 (lower, left panel). Statistical differences were assessed by one-way ANOVA followed by Tukey’s test; different letters indicate significant differences between genotypes (*P* < *0.05*). No correlation was found between meristem size and fully suberized pattern (two-tailed spearman correlation analysis, *P* > 0.05) (lower, right panel). Each dot in the scatter plot represents the mean value for one accession. **d**, Quantification of apoplastic barrier formation in extreme accessions. Violin plots presented as in (**a**) showing the number of cells between the onset of elongation and the site of PI block, *n* = 9 roots for Ven-1 and Kly-1 and *n* = 10 for other accessions (left panel). Statistical analyses were performed as in (**c**). No significant correlation was found between the PI blocking and fully suberized pattern (two-tailed spearman correlation analysis, *P* > 0.05) (right panel). Each dot in the scatter plot represents the mean value for one accession. **a,c,d**, Individual numeric values and the exact *P*-values for the one-way ANOVA and multiple comparisons are provided in Source Data_Extended Data Fig. 1. Source data

**Extended Data Fig. 2:**
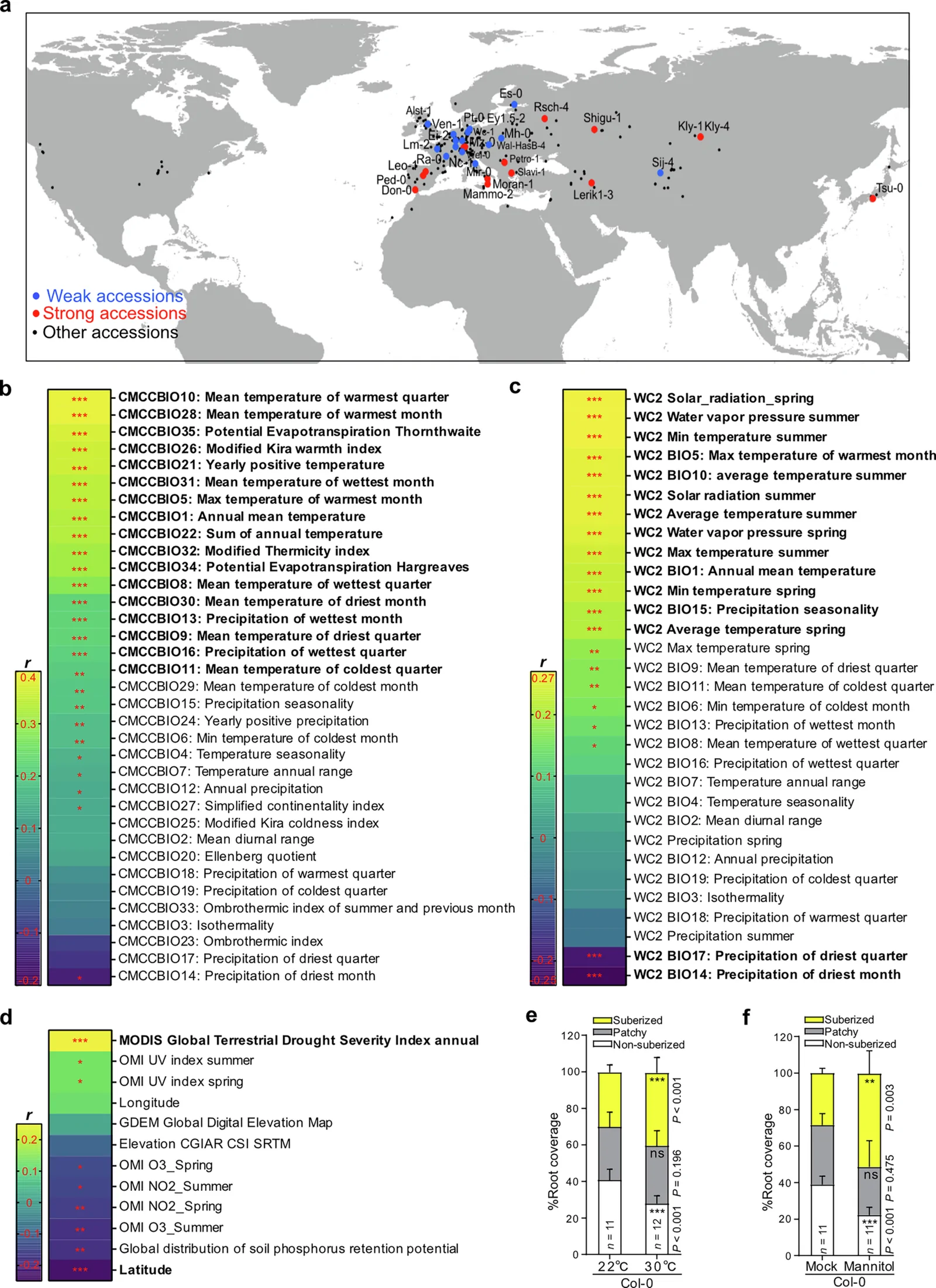
Suberin variations correlate with climate variables. **a**, Geographical origin of the 284 accessions. Weak and strong accessions are highlighted in blue and red, respectively. **b-d**, Correlations between the extent of the suberized zone in 284 accessions and climate variables obtained from 3 different datasets: CMCC (**b**), WorldClimate (**c**) and OMI (**d**). Climate variables with an absolute correlation coefficient (|r|) ≥ 0.2 are shown in bold. A two-tailed Student’s *t*-test was used to assess significance (*P < 0.05, **P < 0.01, ***P < 0.001). Data for (**b**-**d**) as well as the exact *P-*values are provided in Supplementary Table 2. **e**, **f**, Suberin deposition pattern in Col-0 with or without heat (**e**) or osmotic (**f**) stresses. For the treatments, seedlings were grown on 1/2 MS at 22 °C for 4 days, then they were transferred to either 30 °C or 1/2 MS supplemented with 200 mM mannitol for 24 h. Data are presented as stacked columns showing the mean percentage of each suberization zone +/− SD, with *n* shown on the graph. A two-tailed Student’s *t*-test was used to assess significance for the suberization zones (**P < 0.01, ***P < 0.001, ns, not significant). Individual numeric values and the exact *P*-values are provided in Source Data_Extended Data Fig. 2. Source data

To explore the variation of endodermal suberin deposition in *A. thaliana*, we analysed the suberin pattern in 284 genetically diverse natural accessions. We stained 4-day-old seedlings with fluorol yellow to visualize suberin and quantified endodermal suberization by measuring the root segments that were non-suberized, patchy, or fully suberized. A wide range of suberin patterns was observed, particularly in the fully suberized zone (Fig. 1b and Supplementary Table 1). For further analysis, we selected 15 accessions with a significantly reduced proportion of fully suberized cells compared to Col-0 (hereafter referred to as ‘weak’ accessions) and 15 accessions with a significantly extended fully suberized zone (hereafter referred to as ‘strong’ accessions), as determined by quantitative analysis of suberin deposition patterns (Fig. 1c,d). Root length is known to vary across accessions^14,15^, but we found no significant correlation between root length and the fully suberized zone and only weak correlations with the non-suberized and patchy zones (Extended Data Fig. 1a,b and Supplementary Table 1). Minor meristem size variations in specific accessions (for example, Wei-0, Pt-0 and Lerik1-3) did not correlate with suberin patterns (Extended Data Fig. 1c). The above indicates that the observed differences in suberin pattern are not simply a consequence of differences in root growth rate. Since defects in CS formation are known to cause enhanced suberization^11,16–20^, we additionally measured CS functionality using the apoplastic tracer propidium iodide (PI). We confirmed that CS functionality remained intact across the extreme accessions, ruling out defective CS formation as a driver for early or ectopic suberization (Extended Data Fig. 1d). Taken together, these data point to genetic variation directly affecting endodermal suberization, potentially linked to adaptive responses. The geographical distribution of all accessions, particularly those with extreme suberin deposition levels, was mapped, revealing a trend with strong accessions predominantly located in southern Europe, the Mediterranean region and continental Eurasia (Extended Data Fig. 2a). To further explore potential environmental influences, we analysed correlations between suberin deposition and 473 climate variables (Supplementary Table 2). To account for the population structure, the genotype matrix of accessions was summarized by principal component analysis (PCA) and the first 2 principal components (PCs) were taken as covariates in the linear model for correlation analysis. Several strong positive correlations were identified, including those with temperature during the warmest month, summer solar radiation, precipitation seasonality and the global terrestrial drought index (Extended Data Fig. 2b–d). Consistent with these correlations, endodermal suberization in Col-0 increased following a 24-h exposure to elevated temperatures (from 22 °C to 30 °C) or osmotic stress induced by mannitol treatment, which mimics reduced water availability (Extended Data Fig. 2e,f). Notably, Tsu-0, one of the accessions displaying enhanced suberin deposition in our screen, was reported to be drought tolerant^21^, a trait that may be partly attributable to its increased suberization. Together, these findings support the idea that natural variations in endodermal suberization may reflect adaptation to local environmental conditions, particularly temperature and water availability. However, while our data indicate that these stresses can induce suberization, direct links between enhanced suberin deposition and improved physiological performance under heat or drought stress across accessions remain to be established and represent an important direction for future work.

### Suberin variation is associated with specific mineral imbalance

**Extended Data Fig. 3:**
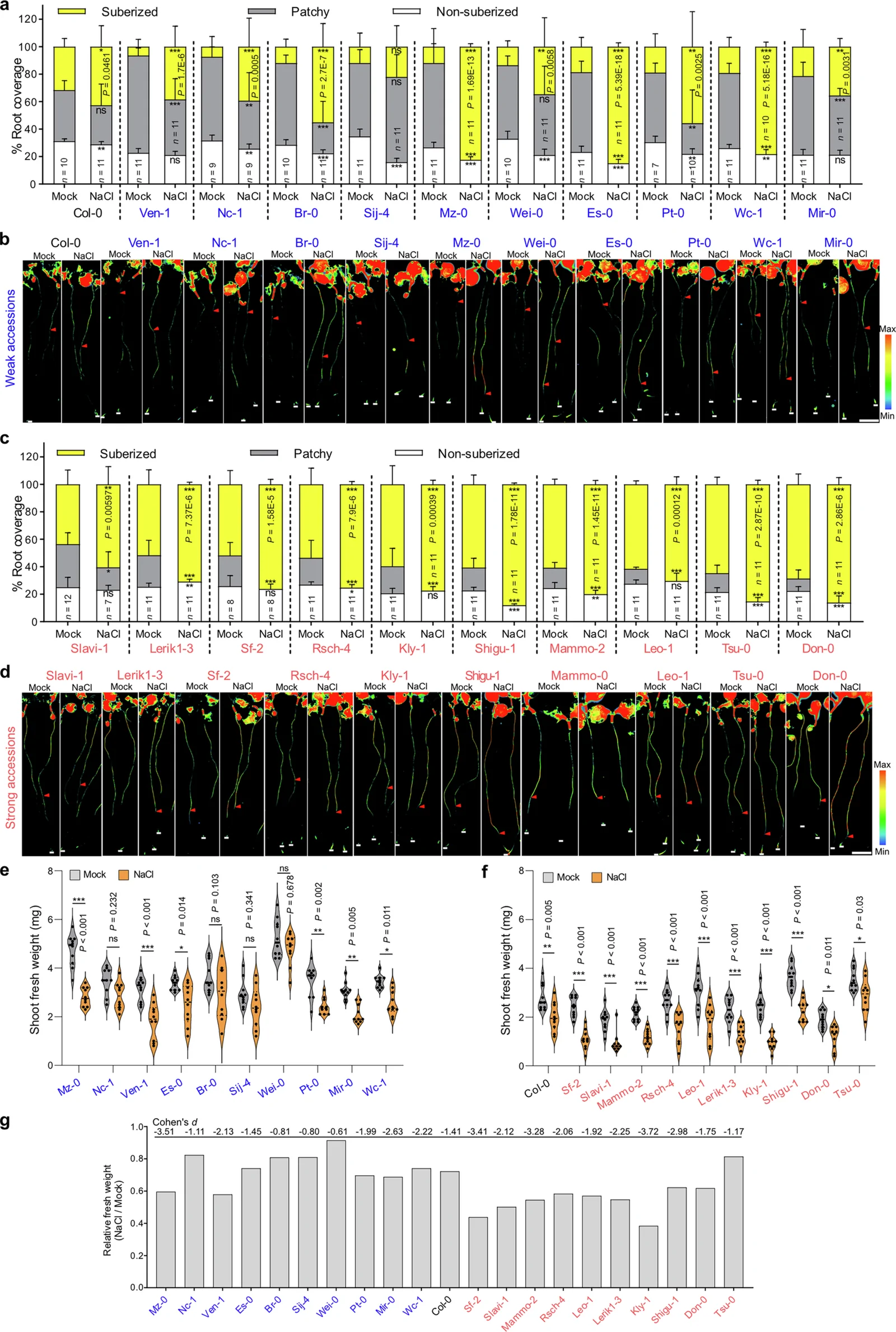
Effect of salt stress on extreme accessions. **a**,**c**, Suberin quantification in Col-0 and 10 weak (**a**) or 10 strong (**c**) accessions under control (mock) or moderate salt stress (85 mM NaCl). 4-day-old seedlings were transferred to mock or NaCl-containing plates for 22 h prior to suberin staining. Data are presented as stacked columns showing the average percentage of each suberization zone +/− SD, with *n* shown on the graphs. Accessions are sorted by percentage of suberized zone under mock condition. Significance between mock and NaCl conditions for the three suberization patterns was tested using a two-tailed Student’s t-test (*P < 0.05, **P < 0.01, ***P < 0.001, ns, not significant). **b**,**d**, Whole-mount images of Fluorol Yellow-stained roots from selected extreme accessions under mock and NaCl conditions from (**a**,**c**). Red arrows indicate the onset of the suberized zone; white lines indicate the root tip. Scale bar, 2 mm. **e**,**f**, Shoot fresh weight of weak (**e**) and strong (**f**) accessions under mock or NaCl conditions. 4-day-old seedlings were transferred to either 1/2 MS plate or 1/2 MS plate supplemented with 85 mM NaCl for 4 days. Data are shown as violin plots with individual values overlaid, *n* = 10. Thick dashed lines in the violin plots indicate the median, while dotted lines represent the first and third quartiles. Two-way ANOVA followed by Sidak’s test was used to assess the significant differences between mock and NaCl conditions for each accession (*P < 0.05, **P < 0.01, ***P < 0.001, ns, not significant). **a,c,e-g**, Individual numeric values and the exact *P*-values are provided in Source Data_Extended Data. Figure 3. **g**, Relative fresh weight of the accessions shown in (**e**,**f**) under mock and NaCl conditions. Cohen’s *d* values are shown on the top of histogram to evaluate the effect sizes by NaCl for each accession. Source data

**Extended Data Fig. 4:**
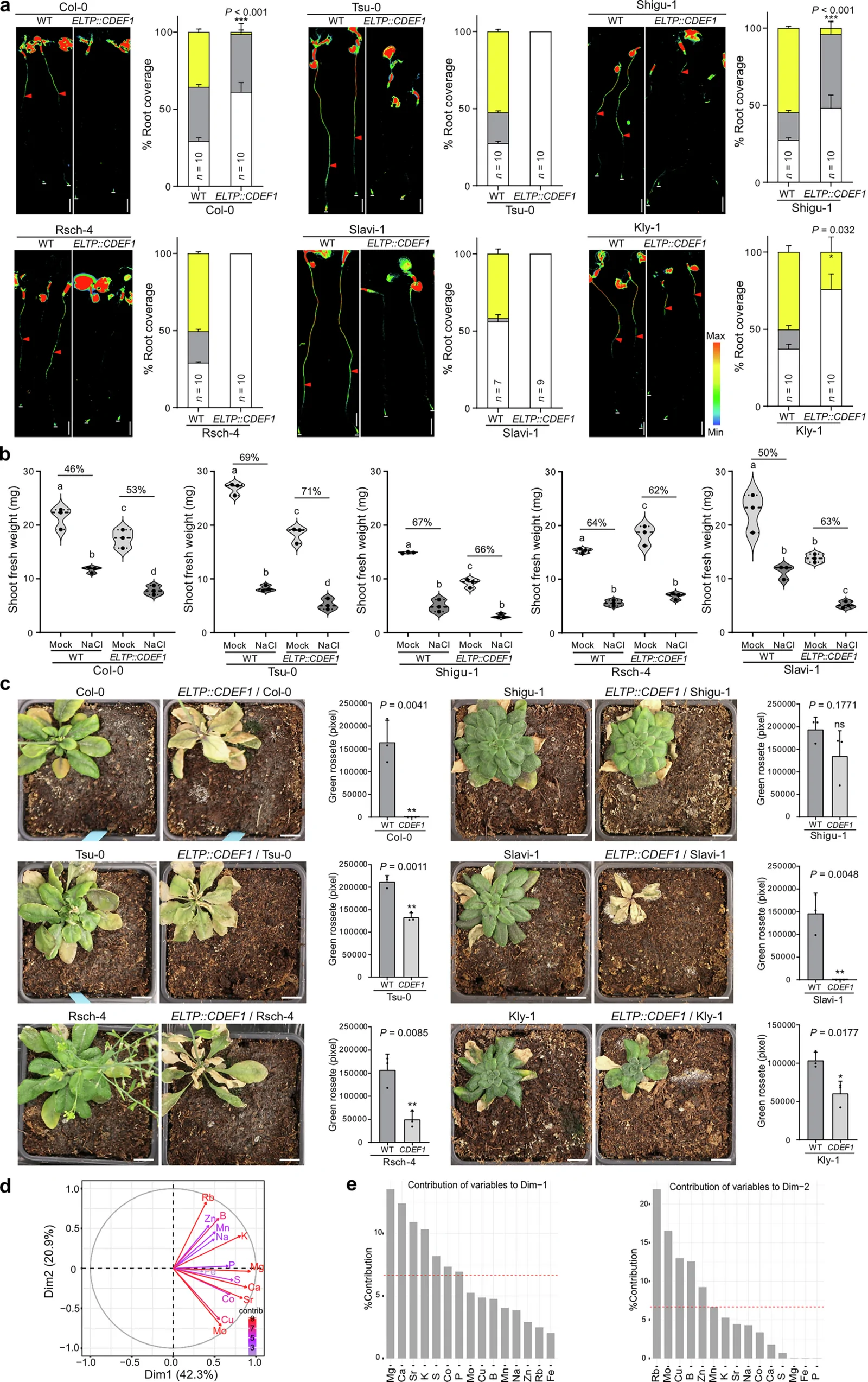
Effect of salt stress on *CDEF1* lines in strong accessions backgrounds. **a**, Whole-mount images of Fluorol Yellow-stained roots and suberin deposition pattern in strong accessions without (WT) or transformed with *ELTP::CDEF1*. Red arrows indicate the onset of suberized zone; white lines mark the root tip. Scale bar, 1 mm. Data are presented as stacked columns showing the mean percentage of each suberization zone +/− SD, with *n* shown on the graphs. A two-tailed Student’s *t-*test was used to assess significant differences for the suberized zone (***P < 0.001, *P < 0.05). **b**, Shoot fresh weight of seedlings grown on 1/2 MS plate for 4 days and then transferred to either 1/2 MS plates or 1/2 MS plates supplemented with 85 mM NaCl for 10 days. Data are shown as violin plots with individual values overlaid, *n* = 3 with each individual value corresponding to the average value for 10 shoots. Thick dashed lines in the violin plots indicate the median, while dotted lines represent the first and third quartiles. One-way ANOVA followed by a Tukey’s test was used to assess significance; different letters indicate significant differences between conditions or genotypes (*P* < 0.05). The reduction ratios of fresh weight by NaCl treatment are presented on the graphs. **c**, Rosette phenotype of plants grown 17 days and then watered with tap water supplemented with 200 mM NaCl for 28 days. Histogram shows the quantification of green rosette size, mean +/− SD *n* = 3 plants. A two-tailed Student’s *t*-test was used to test significant differences between WT and *CDEF1* line (**P < 0.01, *P < 0.05, ns, not significant). Scale bar, 1 cm. **a,b,c**, Individual numeric values and the exact *P*-values are provided in Source Data_Extended Data Fig. 4. **d**, Trait contribution of individual elements to Dim1 and Dim2 from the PCA shown in Fig. 1d. Arrow length and color indicate each element’s contribution to the corresponding principal component. **e**, Histogram of trait contributions to Dim1 and Dim2. The red line indicates the significance threshold above which a trait is considered a major contributor to the respective dimension. Source data

We leveraged the extreme accessions to investigate suberin function in nutrient homeostasis, a process extensively studied in Col-0 (refs. ^2,3,8,13^). Given that in Col-0, salt stress induces endodermal suberization and that suberin acts as barrier to limit sodium (Na) entry^2,3,22,23^, we first assessed suberin induction under moderate salt stress conditions, under which Col-0 displays only a mild response (Extended Data Fig. 3a–d). In this context, all weak accessions showed an increase in suberin, although to varying degrees. Notably, Sij-4, Wei-0 and Mir-0 exhibited minimal induction, indicating a reduced sensitivity to salt-triggered suberization (Extended Data Fig. 3a,b). In contrast, most strong accessions, except Slavi-1, became fully suberized, with Shigu-1, Tsu-0 and Don-0 showing a strong reduction of the non-suberized zone, consistent with a hypersensitivity response (Extended Data Fig. 3c,d). Growth assays further revealed that several weak accessions maintained shoot biomass under salt stress, whereas most strong accessions displayed severe growth reduction (Extended Data Fig. 3e–g). To compare the magnitude of salt effects across accessions, we calculated Cohen’s *d* for the change in shoot biomass between control and salt conditions. All accessions exhibited negative effect sizes (|*d*| = 0.61–3.72), reflecting reduced biomass upon salt treatment. The magnitude of this reduction varied substantially among genotypes: Col-0 showed an intermediate effect (|*d*| = −1.41), similar to the weak accessions Nc-1, Es-0 and Pt-0 and the strong accessions Leo-1, Don-0 and Tsu-0 (|*d*| = 1.11–1.99). The smallest effects (|*d*| = 0.61–0.81) were observed in three weak accessions (Wei-0, Sij-4 and Br-0), whereas the strongest effects (|*d*| = 3.24–3.72) were restricted to one weak accession (Mz-0) and three strong accessions (Mammo-2, Sf-2 and Kly-1). These patterns reveal accession-specific differences in salt sensitivity with a partial separation between weak and strong suberizing accessions. These results contrast with the established model from Col-0 in which reduced suberin increases salt sensitivity and enhanced suberin confers protection^2,8^. To test this further, we expressed *CUTICLE DESTRUCTING FACTOR1* (*CDEF1*) under the *ELTP* promoter to specifically degrade endodermal suberin in strong accessions (Extended Data Fig. 4a), as previously demonstrated in Col-0 (refs. ^2,24,25^), and examined their response to salt stress in plates and soil (Extended Data Fig. 4b,c). Consistent with previous reports, *ELTP::CDEF1* expression in Col-0 increased salt sensitivity, as shown by a stronger reduction in shoot biomass in both growth conditions and a light-brown discolouration. A similar effect was observed in the strong accession Slavi-1, which already differed from other strong accessions in its suberin response to salt. In contrast, suberin degradation in all other strong accessions tested did not alter their salt sensitivity on plates and only led to a mild growth reduction and light-brown discolouration in soil, further supporting that suberin functions in an accession-dependent manner in salt stress responses, distinct from the model established in Col-0.

**Extended Data Fig. 5:**
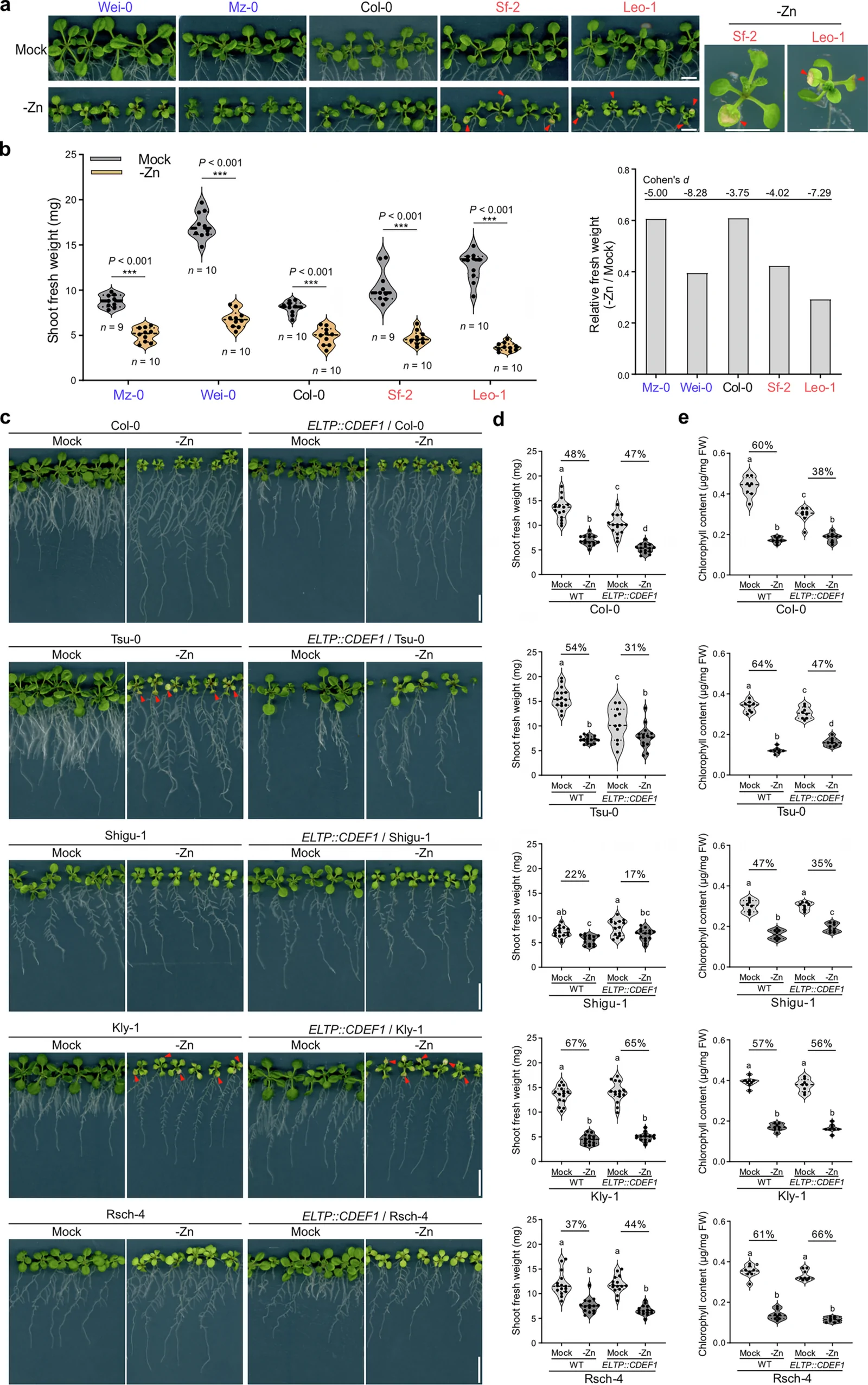
Effect of zinc deficiency on extreme accessions. **a**, Shoot phenotype of 14-day-old extreme accessions grown under control (mock) or Zn-deficient conditions. Enlarged view of Sf-2 and Leo-1 under Zn deficiency, highlights the presence of chlorotic spots (red arrows). Scale bars, 0.5 cm. **b**, Shoot fresh weight of extreme accessions grown under mock or Zn-deficient conditions for 14 days. Data are shown as violin plots with individual values overlaid, with *n* shown on the graph. Thick dashed lines in the violin plots indicate the median, while dotted lines represent the first and third quartiles. Two-way ANOVA was performed followed by Bonferroni’s test to assess significance between treatments for each accession (***P < 0.001). Relative shoot fresh weight under mock and Zn-deficient conditions (right panel) is presented to compare the difference and Cohen’s *d* values are shown on the top of histogram to evaluate the effect sizes by -Zn for each accession. **c**, 14-day-old seedlings of specified accessions without or with *ELTP::CDEF1* constructs (as described in Extended Data Fig. 4a) grown under mock or -Zn conditions. Scale bar, 1 cm. Red arrows indicate chlorotic spots under -Zn. **d**, Shoot fresh weight of seedlings from (**c**). Violin plots presented as in (**b**) showing shoot fresh weight in specified genotypes and conditions, *n* = 15 except for *ELTP::CDEF1/*Tsu-0 in Mock where *n* = 11. One-way ANOVA followed by a Tukey’s test was used to assess significant differences between genotypes; different letters indicate significant differences between conditions or genotypes (*P* < 0.05). The reduction ratios of fresh weight by -Zn condition are presented on the graphs. **e**, Chlorophyll contents of seedlings from (**c**). Data are presented and statistically analyses as in (**d**), *n* = 8. **b,d,e**, Individual numeric values and the exact *P*-values are provided in Source Data_Extended Data Fig. 5. Source data

To further explore the link between suberin variation and mineral homeostasis beyond Na, we conducted ionomic profiling of shoots from 20 extreme accessions alongside Col-0 grown on agar plates. Principal component and hierarchical clustering revealed distinct ionomic signatures between weak and strong accessions, particularly for zinc (Zn), boron (B), rubidium (Rb), copper (Cu) and molybdenum (Mo), but not for Na (Fig. 1e,f, Extended Data Fig. 4d,e and Supplementary Table 3). These results highlight the pivotal role of endodermal suberization in mineral acquisition and confirm trends observed in suberin-altered Col-0 mutants and lines in similar growth conditions. Notably, B was previously shown to accumulate at lower levels in Col-0 plants with ectopic endodermal suberin and at higher levels in those with reduced suberin^3,18^. Similarly, Rb levels were reduced, while Cu and Mo levels increased in Col-0 plants with enhanced suberization^3,13,18^. However, our data also reveal differences from previous findings: Na, which typically accumulates in suberin-deficient Col-0 plants, did not vary significantly here, while Zn, lower in strong accessions in our study, has not previously been identified as a hallmark of suberin-dependent ionomic shifts. We therefore examined whether the lower Zn content observed in strong accessions might be accompanied by altered physiological responses to Zn deficiency. Under Zn-limiting conditions, the strong accessions Sf-2 and Leo-1, as well as the weak accession Wei-0, displayed a pronounced reduction in shoot biomass (Extended Data Fig. 5a,b). To compare the magnitude of Zn deficiency effects among accessions, we calculated Cohen’s *d* for the change in shoot biomass between control and −Zn conditions. All accessions exhibited large negative effect sizes (|*d*| = 3.75–8.28), consistent with a strong reduction in growth under Zn limitation. However, the effect size varied between genotypes, with Wei-0 and Leo-1 showing the largest reduction (|*d*| < −7) and Col-0 the smallest (|*d*| = −3.75), revealing accession-specific differences in Zn sensitivity without a clear separation between the weak and strong accessions analysed. In addition, the strong accessions Sf-2 and Leo-1 developed characteristic Zn-deficiency symptoms in leaves, a phenotype not observed in Col-0 or in the weak accessions Mz-0 and Wei-0 (Extended Data Fig. 5a,b). To clarify whether suberin contributes to these differences, we expressed *ELTP::CDEF1* to degrade suberin in several strong accessions, as described above for salt stress (Extended Data Figs. 4a and 5c–e). In Col-0, suberin degradation had no effect on shoot biomass under Zn limitation but mitigated chlorophyll loss compared to wild type (WT). No significant effect of *ELTP::CDEF1* expression was observed in Kly-1 and Rsch-4. However, in Tsu-0 and Shigu-1, suberin degradation alleviates the negative effect of Zn limitation on both shoot biomass and chlorosis. Together, these results demonstrate an accession-dependent role of endodermal suberin in Zn, neutral in some accessions but exacerbating Zn sensitivity in others. They reveal a previously underappreciated role of endodermal suberization in Zn acquisition and highlight a trade-off between protective barrier function and micronutrient uptake. While they challenge the view of suberin as a strict protective barrier against Na, our findings underscore its impact on Zn homeostasis, revealing that increased suberin deposition correlates with reduced Zn accumulation and heightened sensitivity to Zn limitation in specific genetic backgrounds.

### GWAS for endodermal suberin deposition identifies *SBG1*

**Fig. 2:**
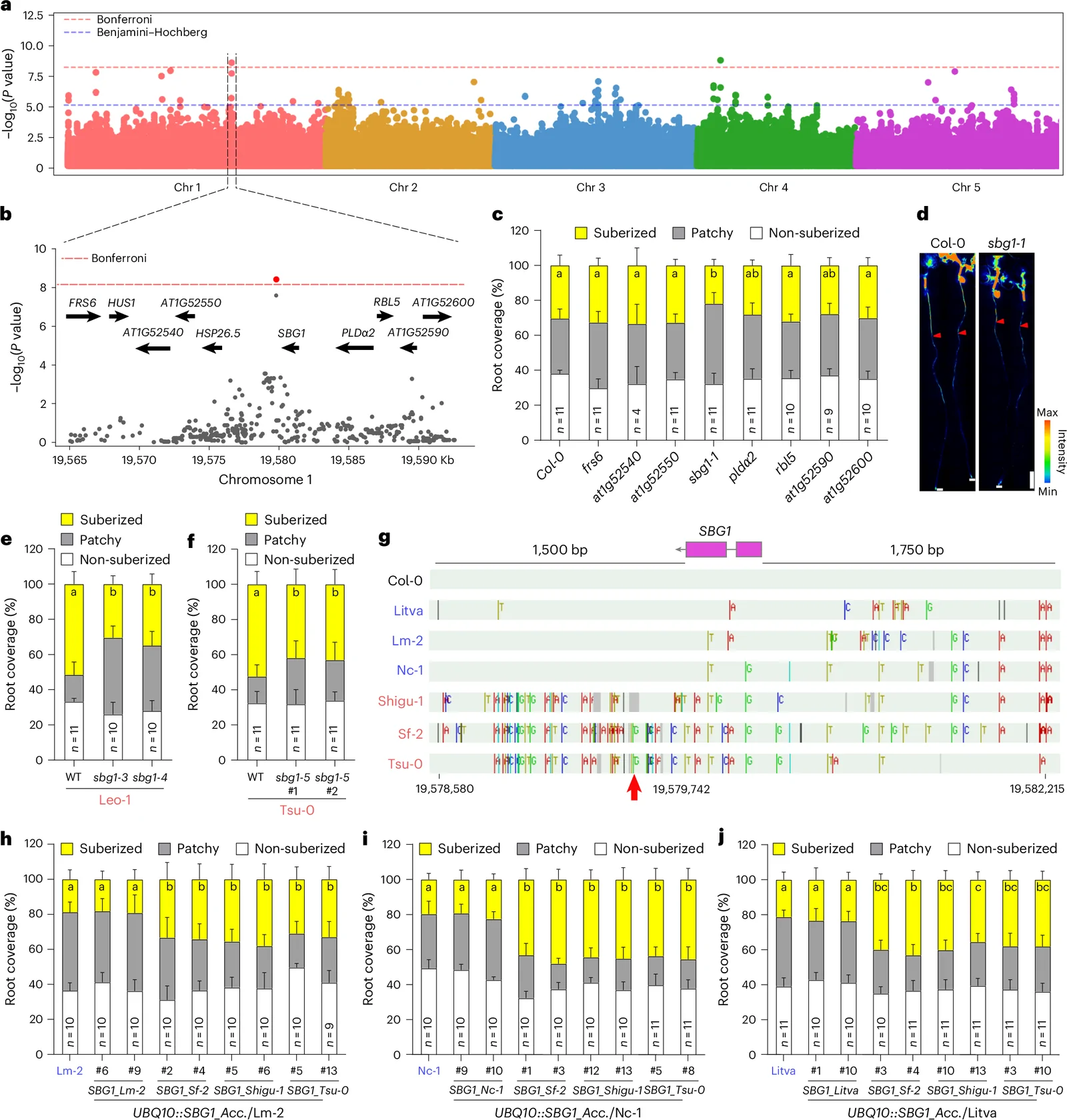
Identification of *SBG1* as a genetic determinant of suberin variations. **a**, Manhattan plot of GWAS for suberin variations across 265 accessions. Horizontal dashed lines indicate significant thresholds after multiple testing correction: red for Bonferroni and blue for Benjamini–Hochberg (FDR = 10%). **b**, SNPs and annotated genes surrounding the *SBG1* genomic region. Each dot represents an SNP, with the top SNP (19,579,742 bp) highlighted in red. AMM employed a parametric *F*-test to obtain the *P* values. **c**, Suberin deposition pattern in T-DNA insertion lines for candidate genes shown in **b**. Data are presented as stacked columns showing the mean percentage of each suberization zone ±s.d., with *n* shown on the graphs. One-way ANOVA followed by Tukey’s test was used to assess significance for the suberized zone; different letters indicate significant differences between genotypes (*P* < 0.05). **d**, Whole-mount images of fluorol yellow-stained roots from Col-0 and *sbg1-1* shown in **c**. Red arrowheads indicate the onset of the suberized zone; white lines mark the root tip. Scale bar, 1 mm. **e**,**f**, Suberin deposition pattern in *sbg1* mutants generated in Leo-1 (**e**) and Tsu-0 (**f**) backgrounds. Data are presented as in **c**. One-way ANOVA followed by Tukey’s test was used to test significance of deposition differences in the suberized zone (*P* < 0.05). **g**, SNP profiles in the *SBG1* genomic region across 3 *T* and 3 *G* allele accessions. The top SNP from GWAs is marked with a red arrow. SNP data were obtained from the 1001 Genomes dataset. **h**–**j**, Suberin deposition patterns in transgenic lines expressing *SBG1* under the *UBQ10* promoter in the background of Lm-2 (**h**), Nc-1 (**i**) and Litva (**j**). *SBG1* genomic sequence including 1.5 kb downstream of the stop codon was derived from Lm-2, Nc-1, Litva, Sf-2, Shigu-1 or Tsu-0 as indicated. Two independent lines were analysed per construct. Data are presented as in **c**. One-way ANOVA followed by Tukey’s test was used to determine significance of differences in the suberized zone (*P* < 0.05). **c**,**e**,**f**,**h**–**j**, Individual numeric values and the exact *P* values for multiple comparisons are provided in Source Data Fig. 2. Source data

**Extended Data Fig. 6:**
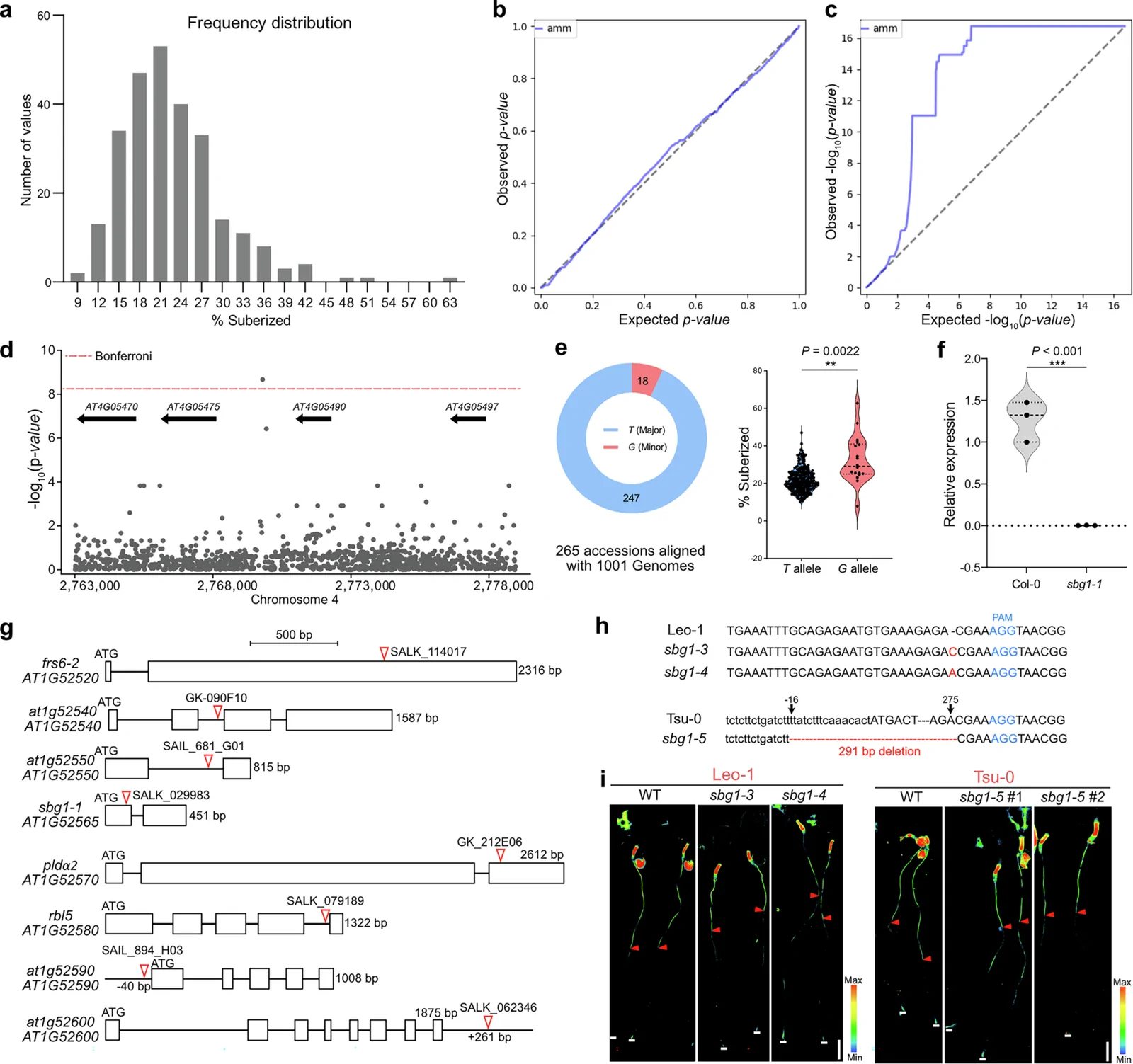
Mutations of *SBG1* in strong accessions reduces suberin. **a**, Frequency distribution of the fully suberized zone across 265 *Arabidopsis* accessions used for the GWAS shown in Fig. 2a. **b**,**c**, Quantile-quantile (QQ) plots corresponding to the GWAS shown in the Manhattan plot in Fig. 2a. **d**, SNPs located at positions 2,769,796 and 2,769,807 on Chromosome 4. AMM employed a parametric *F*-test to obtain the P values. **e**, Percentage of the suberized zone in accessions carrying the 2 alleles at SNP position 19,579,742 on Chromosome 1. Data are presented as violin plots with outlier values overlaid. Thick dashed lines in the violin plots indicate the median, while dotted lines represent the first and third quartiles. A two-tailed Student’s *t-*test was performed, **P < *0.01* (details provided on Source Data_Extended Data Fig. 6) **f**, RT-qPCR analysis of *SBG1* relative transcript levels in roots of Col-0 and *sbg1-1*. Violin plots presented as in (**e**) showing the RT-qPCR results, *n* = 3. A two-tailed Student’s t-test was performed, ***P < 0.001. Individual numeric values and details provided in Source Data_Extended Data Fig. 6. **g**, Schematic showing the position of the T-DNA in the gene candidates for which mutants were characterized in Fig. 2c. Homozygous mutants were isolated by genotyping and T-DNA insertion sites were confirmed by sequencing. **h**, Schematic showing the position of CRISPR-generated mutations for *SBG1* in Leo-1 and Tsu-0 backgrounds. **i**, Whole-mount Fluorol Yellow-stained roots of *sbg1* mutants in Leo-1 and Tsu-0 backgrounds. Red arrows indicate the onset of the suberized zone; white lines mark the root tip. Scale bar, 1 mm. Source data

To uncover the genetic basis of natural variation in endodermal suberin deposition, we performed a GWAS for the percentage of the fully suberized zone (Fig. 1b, Extended Data Fig. 6a–c and Supplementary Table 1)^26^. The broad-sense heritability (*H*^2^) for the suberized zone was estimated to be 0.55, indicating a substantial genetic contribution to variation in endodermal suberization while also reflecting the influence of environmental and measurement variance. GWAs identified two significant loci on chromosomes 1 and 4 (Fig. 2a) with the lead single nucleotide polymorphism (SNP) (*G*/*T*) on chromosome 1 located 319 bp downstream of *AT1G52565*, a previously uncharacterized gene encoding a 129-amino-acid protein, later designated SUBER GENE1 (SBG1) (Fig. 2b). On chromosome 4, two SNPs mapped between *AT4G05475* and *AT4G05490* encoding putative F-BOX proteins, but given their greater distance from the SNPs (1,839 bp and 1,236 bp, respectively) (Extended Data Fig. 6d), we focused on the chromosome 1 locus. Accessions carrying the *G* allele at this locus exhibited significantly higher suberin levels than those with the *T* allele (Extended Data Fig. 6e).

To validate the role of *SBG1*, we quantified suberin deposition in available T-DNA insertion lines in Col-0 for genes within ±15 Kb of the lead SNP (Fig. 2c). Only the *sbg1-1* mutant showed significantly reduced endodermal suberin deposition compared to WT (Fig. 2c,d and Extended Data Fig. 6f). This phenotype was independently confirmed in CRISPR-Cas9-generated *sbg1* mutants in the strong accessions Leo-1 and Tsu-0, further supporting the role of *SBG1* in suberization (Fig. 2e,f and Extended Data Fig. 6h,i). To explore how allelic variation influences suberization, we compared *SBG1* sequences between weak (*T* alleles) and strong (*G* alleles) accessions. Protein alignment revealed a 4-amino-acid insertion (REQR in place of S83) in the strong alleles, but structural prediction using AlphaFold did not indicate any effect on protein folding that would allow us to formulate a hypothesis on a functional effect of this insertion (Supplementary Fig. 1a,b). Sequence comparison of *T* and *G* alleles revealed several additional SNPs clustered in the 3′ region (Fig. 2g). Because 3′ UTRs often regulate messenger (m)RNA stability and translation, we hypothesized that these polymorphisms could affect *SBG1* expression. However, quantitative PCR with reverse transcription (RT–qPCR) analysis did not reveal a consistent correlation between *SBG1* transcript levels and suberization across accessions (Supplementary Fig. 1c). Interestingly, secondary structure predictions for the 3′UTR showed pronounced differences between *T* and *G* alleles (Supplementary Fig. 2a,b), suggesting potential regulatory effects that merit further investigation. To functionally assess allele-specific effects, we cloned *SBG1* genomic sequences, including 1.5 kb of the 3′ region, from accessions carrying either the *G* allele (strong accessions, Shigu-1, Sf-2 and Tsu-0) or the *T* allele (weak accessions Lm-2, Nc-1 and Litva). When expressed under the constitutive and ubiquitous *UBQ10* promoter in *T* allele backgrounds (Lm-2, Nc-1 and Litva), *T* allele transgenes did not alter suberization, whereas *G* allele constructs significantly enhanced suberin deposition near the root tip (Fig. 2h–j). Together, these findings establish *SBG1* as a key positive regulator of suberization across natural accessions, demonstrating that allelic variations in *SBG1* modulate suberin deposition in the root endodermis.

### *SBG1* is a positive regulator of endodermal suberization

**Fig. 3:**
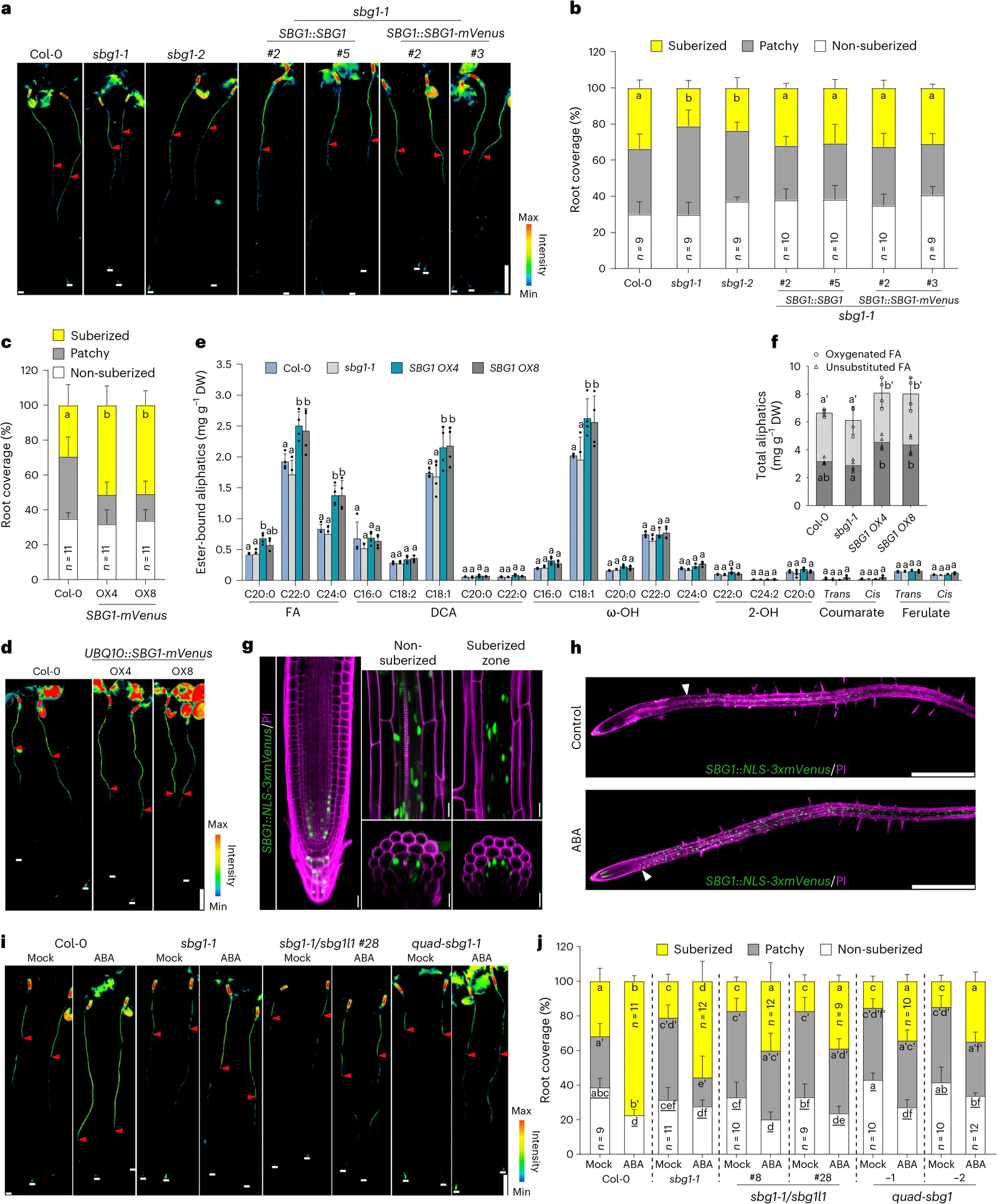
*SBG1* regulates suberization. **a**,**b**, Whole-mount images of fluorol yellow-stained roots (**a**) and suberin deposition pattern (**b**) in *sbg1* mutants and complemented lines. Red arrowheads indicate the onset of the suberized zone; white lines mark the root tip. Scale bar, 1 mm. Data are presented as stacked columns showing the mean percentage of each suberization zone ±s.d., with *n* shown on the graphs. One-way ANOVA followed by Tukey’s test was used to assess significance for the suberized zone; different letters indicate significant differences between genotypes (*P* < 0.05). **c**,**d**, Whole-mount images (**d**) and quantification (**c**) of suberin deposition in transgenic lines expressing *SBG1-mVenus* under the *UBQ10* promoter. Data are presented as in **a** and **b**. **e**,**f**, Quantification of ester-bound aliphatic and aromatic (Coumarate and Ferulate) suberin monomers (**e**) and the corresponding total amount of unsubstituted and oxygenated fatty acids (**f**) in 5-day-old roots. FA, fatty acid; DCA, dicarboxylic fatty acid; ω-OH, ω-hydroxy fatty acid; DW, dry weight. Data are shown as histograms with individual values overlaid, mean ± s.d., *n* = 4 (pools of 200–300 roots). Two-way ANOVA followed by Tukey’s test was used to assess significance for each suberin monomer, oxygenated and unsubstituted FA; different letters indicate significant differences between genotypes (*P* < 0.05). **g**, Confocal images of *SBG1::NLS-3xmVenus* line showing mVenus signal (green) combined with PI staining (magenta) to highlight cell walls. In the non-suberized and suberized root zones, mVenus signal was projected in 3D. Cross-section views were generated by reslicing *Z*-stacks. Scale bar, 20 µm. **h**, Confocal images of *SBG1::NLS-3xmVenus* lines treated with or without 1 µM ABA for 18 h. mVenus signal is shown in green and PI in magenta. White arrowheads indicate the onset of mVenus signal after the division zone. Scale bar, 500 µm. **g**,**h**, Experiment was repeated 3 times independently with similar results. **i**,**j**, Whole-mount images (**i**) and suberin deposition pattern (**j**) in *sbg1-1* and high-order *sbg1l* mutants under control (mock) or 1 µM ABA treatment for 18 h. Seedlings (4-day-old) were transferred to either 1/2 MS plates or 1/2 MS plate + ABA. Data are presented as in **a** and **b**. Different letters indicate significant differences in the different suberized zones between genotypes and conditions, with apostrophes and underlines for the patch and non-suberized zones, respectively. **b**,**c**,**e**,**f**,**j**, Individual numeric values and the exact *P* values for multiple comparisons are provided in Source Data Fig. 3. Source data

**Extended Data Fig. 7:**
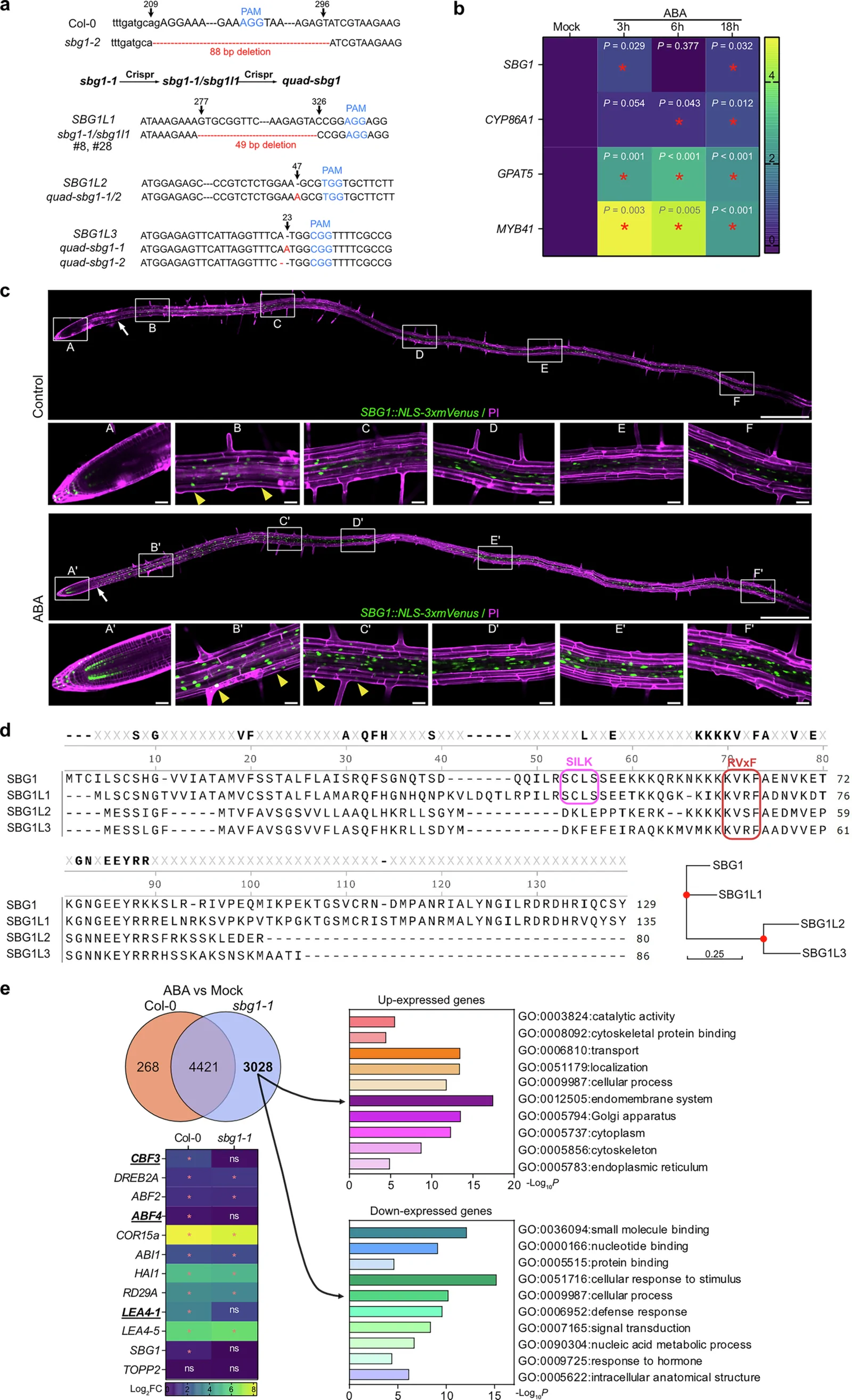
ABA induces the expression of *SBG1*. **a**, Schematic representation of the CRISPR-generated mutation in *SBG1* and its homologs to generate the *sbg1-2*, *sbg1-1;sbg1l1* double mutant and *quad-sbg1* mutants in the Col-0 background. **b**, Heat map of qRT-PCR analysis showing relative transcript levels of *SBG1*, *CYP86A1/HORST*, *GPAT5* and *MYB41* under control (Mock) or 1 µM ABA treatment for 3, 6, or 18 h. Data presented as log_2_(fold change) relative to mock. One-tailed Student’s *t*-test was used to assess significance between control and ABA treatments at each time point (*P < 0.05, *n* = 3). Individual numeric values and the exact *P*-values are provided in Source Data_Extended Data Fig. 7. **c**, Confocal images of *SBG1::NLS-3xmVenus* lines treated with or without 1 µM ABA for 18 h. The full scans are identical to Fig. 4h and additional higher-resolution images for positions specified with a white box are presented. mVenus signal is shown in green and PI in magenta. White arrows indicate the onset of mVenus signal after the division zone. Yellow arrows indicate signal in the cortex or epidermis. Scale bar, 500 µm. Experiment was repeated 3 times independently with similar results. **d**, Protein sequence alignment and phylogenetic analysis of SBG1, SBG1L1, SBG1L2 and SBG1L3. The SILK and RVxF motifs, known to mediate binding to TOPPs, are highlighted in pink and red respectively. On the phylogenetic tree, each red node represents a divergence event. **e**, Comparison of differentially expressed genes (DEG) from RNAseq in Col-0 and *sbg1-1* roots after 2 µM ABA treatment for 4 h. Heat maps showing the differential expression of ABA-induced maker genes, *SBG1* and *TOPP2* in Col-0 and *sbg1-1* (*Padj < 0.05, ns, not significant, *n* = 3 pools of approximately 250 roots). GO enrichment analysis was performed for the 3028 unique DEGs in *sbg1-1*. Source data

To further investigate the role of SBG1 in suberin deposition, we functionally characterized this gene in Col-0. The delayed pattern of endodermal suberin deposition observed by histochemical staining in *sbg1-1* (Fig. 2c,d) was confirmed with a second allele, *sbg1-2*, generated by CRISPR-Cas9 (Fig. 3a,b and Extended Data Fig. 7a). Complementation of *sbg1-1* with either SBG1 or SBG1-mVenus under its native promoter fully restored suberization (Fig. 3a,b). Conversely, constitutive expression of SBG1-mVenus under the *UBQ10* promoter significantly enhanced suberization near the root tip (Fig. 3c,d), consistent with the enhanced suberization observed when *G* alleles were expressed in weak accessions (Fig. 2h–j). Although no significant difference in total suberin content was detected in *sbg1-1* mutants compared to Col-0, the enhanced suberization in *UBQ10::SBG1-mVenus* lines was corroborated by chemical analysis of whole-root suberin content, revealing a significant increase in suberin monomers, with a 34% increase in total aliphatic content (Fig. 3e,f). Of note, the most abundant monomers were particularly increased—C22:0 and C24:0 fatty acids, C18:1 dicarboxylic acid (DCA), and C18:1 *ω*-hydroxy fatty acid, corresponding to the main monomers also increased after ABA application^2^ or after *MYB41* endodermal overexpression^3^. Together, these results demonstrate that *SBG1* positively regulates endodermal suberin deposition. A defining feature of genes involved in endodermal suberin formation is their specific expression in this tissue and their induction by ABA^2,3,6,7,12,27^. Whole-root expression analysis revealed that *SBG1* was moderately upregulated after ABA treatment (Extended Data Fig. 7b), reaching levels comparable to that of *CYP86A1* (also known as *HYDROXYLASE OF ROOT SUBERIZED TISSUE* (*HORST*)), a cognate suberin biosynthetic enzyme^28^. Notably, this induction is markedly weaker than that of *MYB41*, a major transcriptional activator of endodermal suberization^3^. Such a moderate increase, while clearly ABA responsive, might reflect a function in suberin biosynthesis or as a fine-tuning component rather than a primary regulator within the endodermal suberin network. To examine *SBG1* expression in situ, we generated *SBG1::NLS-3xmVenus* transcriptional reporter lines. Promoter activity was initially detected in the columella and early endodermal lineage cells at the root tip (Fig. 3g). In differentiated roots, *SBG1* promoter activity became predominantly endodermal, with weaker activity observed in vascular tissues (Fig. 3g). Following ABA treatment, promoter activity expanded into the early pericycle and endodermal cell lineages, and was additionally detected in some epidermal and cortical cells in the elongation and early differentiation zones (Fig. 3h and Extended Data Fig. 7c). Moreover, consistent with suberin-related genes such as *MYB41, MYB92* and *GPAT5* (refs. ^2,3^), ABA further enhanced *SBG1* promoter activity in the endodermis, extending expression towards the meristematic zone (Fig. 3h and Extended Data Fig. 7c).

The *A. thaliana* genome contains three uncharacterized homologues of *SBG1*, hereafter referred to as *SBG1-Like1, SBG1-Like2* and *SBG1-Like3* (*SBG1L1–3*) (Extended Data Fig. 7d). Under control conditions, the double mutant *sbg1;sbg1l1* was phenotypically similar to *sbg1*, while the quadruple mutant *sbg1;sbg1l1;sbg1l2;sbg1l3* (*quad-sbg1*) displayed only a slightly extended non-suberized zone (Fig. 3i,j). Under ABA treatment, however, suberin induction was progressively attenuated from *sbg1* to *sbg1;sbg1l1*, and most strongly in *quad-sbg1*, which exhibited a suberization pattern resembling that of untreated Col-0 roots. These results suggest that SBG1 and its homologues contribute additively but modestly to ABA-mediated suberization response in endodermal cells.

To test this hypothesis, we compared the transcriptional responses of Col-0 and *sbg1* roots to ABA by RNA-seq. Given our hypothesis that *SBG1* acts as a local modulator of ABA response in a limited number of endodermal cells, we expected only subtle global transcriptional effects. Consistent with this, under control conditions, only minor expression differences were detected between *sbg1* and Col-0, with 29 genes upregulated and 45 downregulated, and no enrichment for specific biological processes (Supplementary Table 4). We next compared the ABA-induced genes in Col-0 and *sbg1* and observed striking differences between genotypes, with 4,689 genes affected in Col-0 and 7,449 in *sbg1*, with only 59% shared with Col-0 (Extended Data Fig. 7e and Supplementary Table 4), demonstrating that *sbg1* is affected in its ABA response. Among the 268 genes induced by ABA in Col-0 but not in *sbg1*, we could find *SBG1* as expected as well as several well-known ABA-responsive genes such as *C-REPEAT BINDING FACTOR 3* (*CBF3*), *ABRE BINDING FACTOR 4* (*ABF4*) and *LATE EMBRYOGENESIS ABONDANT 4-1* (*LEA4-1*) (Extended Data Fig. 7e). These results provide strong evidence that *sbg1* is affected in its transcriptional response to ABA.

**Extended Data Fig. 8:**
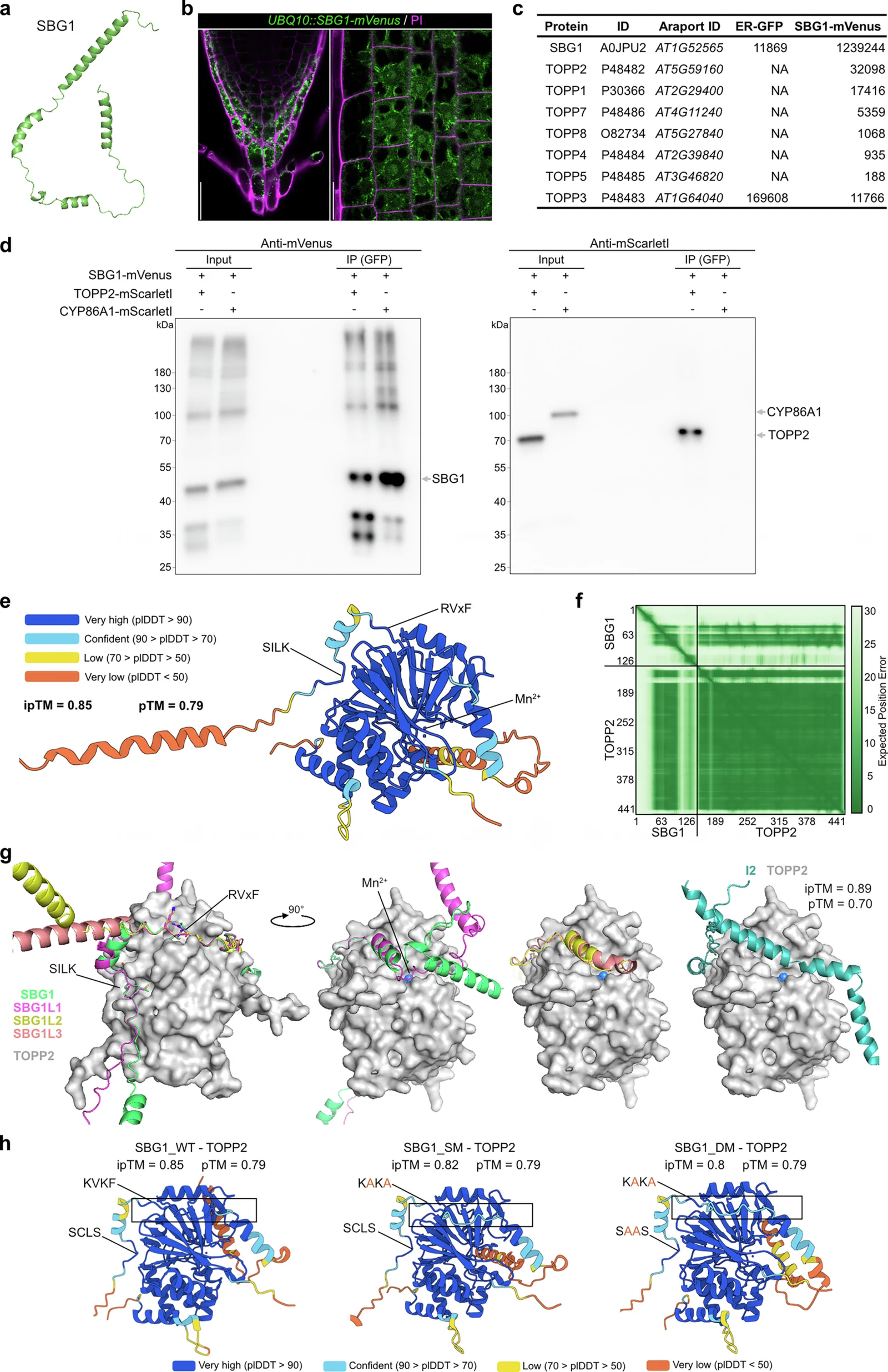
Identification of TOPPs as targets of SBG1s. **a**, Predicted AlphaFold structure of SBG1. **b**, Confocal images of SBG1-mVenus (green) in the *SBG1 OX4* line with PI staining (magenta) to highlight cell walls. Scale bar, 20 µm. Experiment was repeated 3 times independently with similar results. **c**, List of TOPP candidate interactors identified by IP-MS. Peptide quantification is shown for each protein, *n* = 3. Individual numeric values provided in Supplementary Table 5. **d**, Full membrane views of the CoIP assay showed in Fig. 4b. Experiment was repeated 2 times independently with similar results. **e**, Predicted AlphaFold structure of SBG1 in complex with TOPP2. Cartoon representation colored by predicted local distance difference test (pLDDT) confidence scores. **f**, Predicted aligned error (PAE) plot corresponding to the AlphaFold model shown in (**e**). **g**, Aligned AlphaFold structures of SBG1 homologs in complex with TOPP2. Structural differences between SBG1/SBG1L1 and SBG1L2/SBG1L3 are observed at both N- and C- termini (left panels). Predicted AlphaFold structure of Inhibitor 2 (I2) in complex with TOPP2 (right panel). **h**, AlphaFold predicted structure of SBG1_WT, SBG1_SM or SBG1_DM complex with TOPP2. Cartoon color represented as in (**e**). The mutated amino acids are marked in red; black boxes indicate the RVxF region with changed pLDDT score in SBG1_WT, SBG1_SM and SBG1_DM.

Together, these results establish SBG1 and its homologues as positive regulators of ABA-mediated endodermal suberization and reveal that SBG1 contributes to ABA responsiveness in the root. However, given that *SBG1* encodes a small 129-amino-acid protein with no known functional domains, its precise role in ABA response or suberin formation remains elusive (Extended Data Fig. 8a).

### SBG1 promotes suberization through interference with TOPPs

**Fig. 4:**
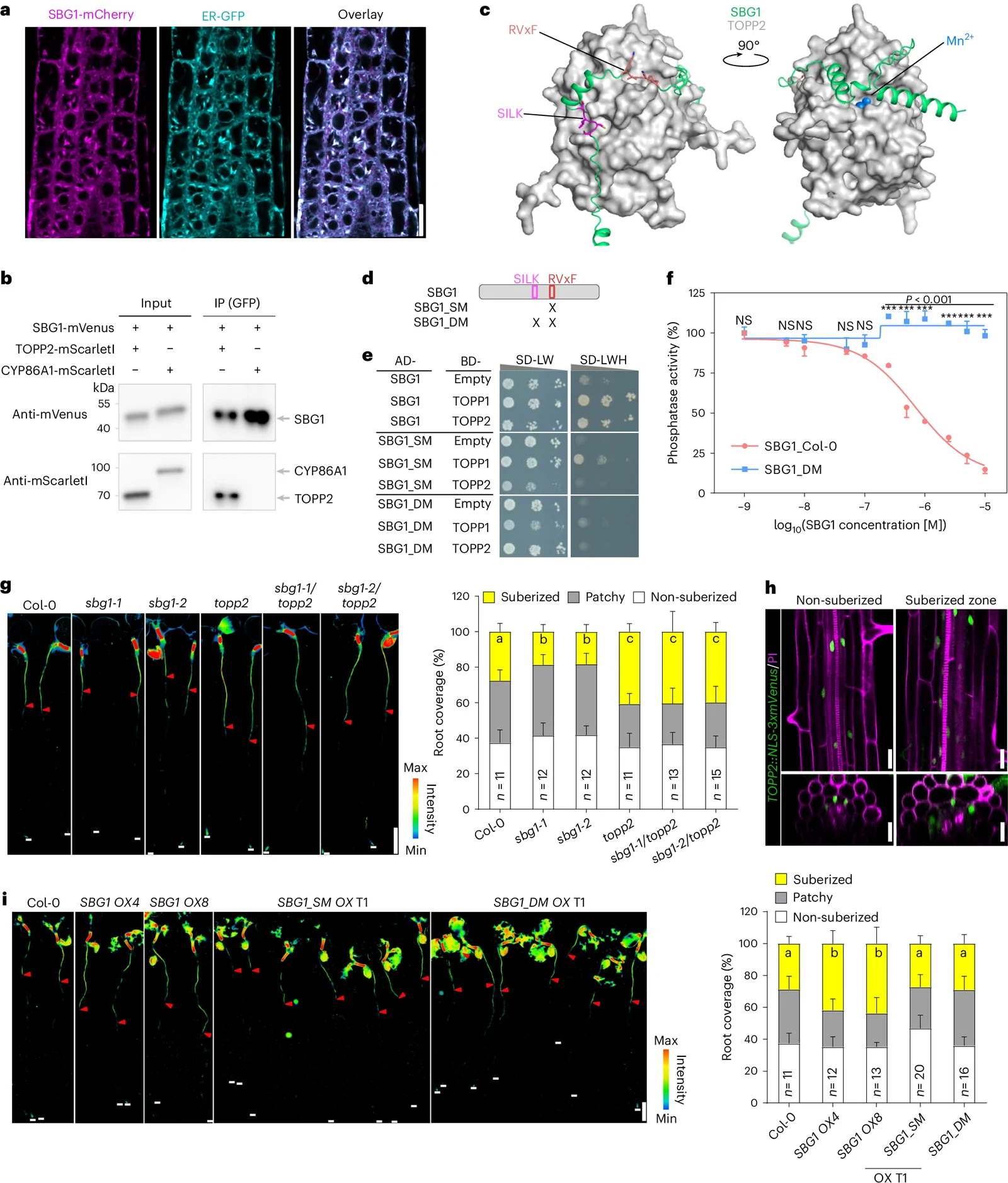
SBG1 regulates suberization through its interaction with TOPPs. **a**, Confocal images showing co-localization of SBG1-mCherry (magenta) with ER-GFP (cyan). Individual channels and merged images are shown. Scale bar, 50 µm. Experiment was repeated 3 times independently with similar results. **b**, Co-IP assay showing SBG1 interaction with TOPP2 in roots from plants co-expressing SBG1-mVenus and TOPP2-mScarletI or SBG1-mVenus and CYP86A1-mScarletI as a negative control. See also Extended Data Fig. 8d. Experiment was repeated 2 times independently with similar results. **c**, Predicted AlphaFold3 structure of SBG1 in complex with TOPP2. The side chains of the SILK and RVxF motifs in SBG1 and 2 Mn^2+^ ions in the catalytic domain of TOPP2 are highlighted. **d**, Schematic representation of mutations in the SBG1 binding motifs. SM denotes mutation in the RVxF motif, and DM denotes combined mutations in both the SILK and RVxF motifs. Mutation sites are marked with an ‘X’. **e**, Yeast two-hybrid assay showing interactions between WT or mutated forms of SBG1 (SM and DM) and TOPP1 or TOPP2. Drop test started with an OD_600_ of 0.1 followed by 10 times dilutions. **f**, Phosphatase activity assay showing SBG1 concentration-dependent inhibition of TOPP2 activity in vitro. Two-way ANOVA followed by Sidak’s test was used to assess the significance at each concentration (***P < 0.001; NS, not significant, *n* = 3). **g**, Whole-mount images of fluorol yellow-stained roots and corresponding suberin deposition pattern in *sbg1*, *topp2* and *sbg1/topp2* double mutants (left panels). Red arrowheads indicate the onset of the suberized zone; white lines mark the root tip. Scale bar, 1 mm. In the right panel, data are presented as stacked columns showing the mean percentage of each suberization zone ±s.d., with *n* shown on the graph. One-way ANOVA followed by Tukey’s test was used to assess significance for the suberized zone; different letters indicate significant differences between genotypes (*P* < 0.05). **h**, Confocal images of *TOPP2::NLS-3xmVenus* in roots with mVenus signal (green) overlaid with PI staining (magenta) to visualize cell walls. mVenus signal was projected in 3D. Cross-section views were generated by reslicing *Z*-stacks. Scale bar, 50 µm. **i**, Whole-mount images and suberin deposition pattern in T1 overexpression lines expressing *SBG1_SM* and *SBG1_DM* compared to WT *SBG1* (*SBG1 OX4* and *8*) (left panels). In the right panel, data are presented and statistically analysed as in **g**. Both *SBG1_SM OX* and *SBG1_DM OX* correspond to independent T1 lines. **f**,**g**,**i**, Individual numeric values and the exact *P* values for two-way ANOVA and multiple comparisons are provided in Source Data Fig. 4. Source data

We hypothesized that SBG1’s molecular function might be regulatory, mediated through protein–protein interactions. We first assessed SBG1 subcellular localization with SBG1-mVenus and SBG1-mCherry fusion proteins, which exhibited a reticulated signal co-localizing with an endoplasmic reticulum (ER) marker (Fig. 4a and Extended Data Fig. 8b). We next performed immunoprecipitation (IP) of SBG1-mVenus from plants constitutively expressing the fusion protein, followed by mass spectrometry (MS) analysis. Enriched proteins were identified by comparison with an IP control from plants expressing ER-GFP. Among the most enriched proteins (Supplementary Table 5), we identified 2 TOPPs: TOPP2 and TOPP1 (Extended Data Fig. 8c). In total, 6 out of the 9 *A. thaliana* TOPPs (TOPP1, 2, 4, 5, 7 and 8) were significantly enriched in the SBG1 IP–MS dataset (Extended Data Fig. 8c). A Fisher’s exact test confirmed this as a highly significant enrichment (*P* = 6.3 × 10^−5^; odds ratio = 15.5), supporting the specificity of the association between SBG1 and TOPP proteins. The interaction between SBG1 and TOPP2, the most strongly enriched phosphatase, was further validated by co-immunoprecipitation in planta, using seedlings co-expressing SBG1-mVenus and TOPP2-mScarletI (Fig. 4b and Extended Data Fig. 8d).

**Extended Data Fig. 9:**
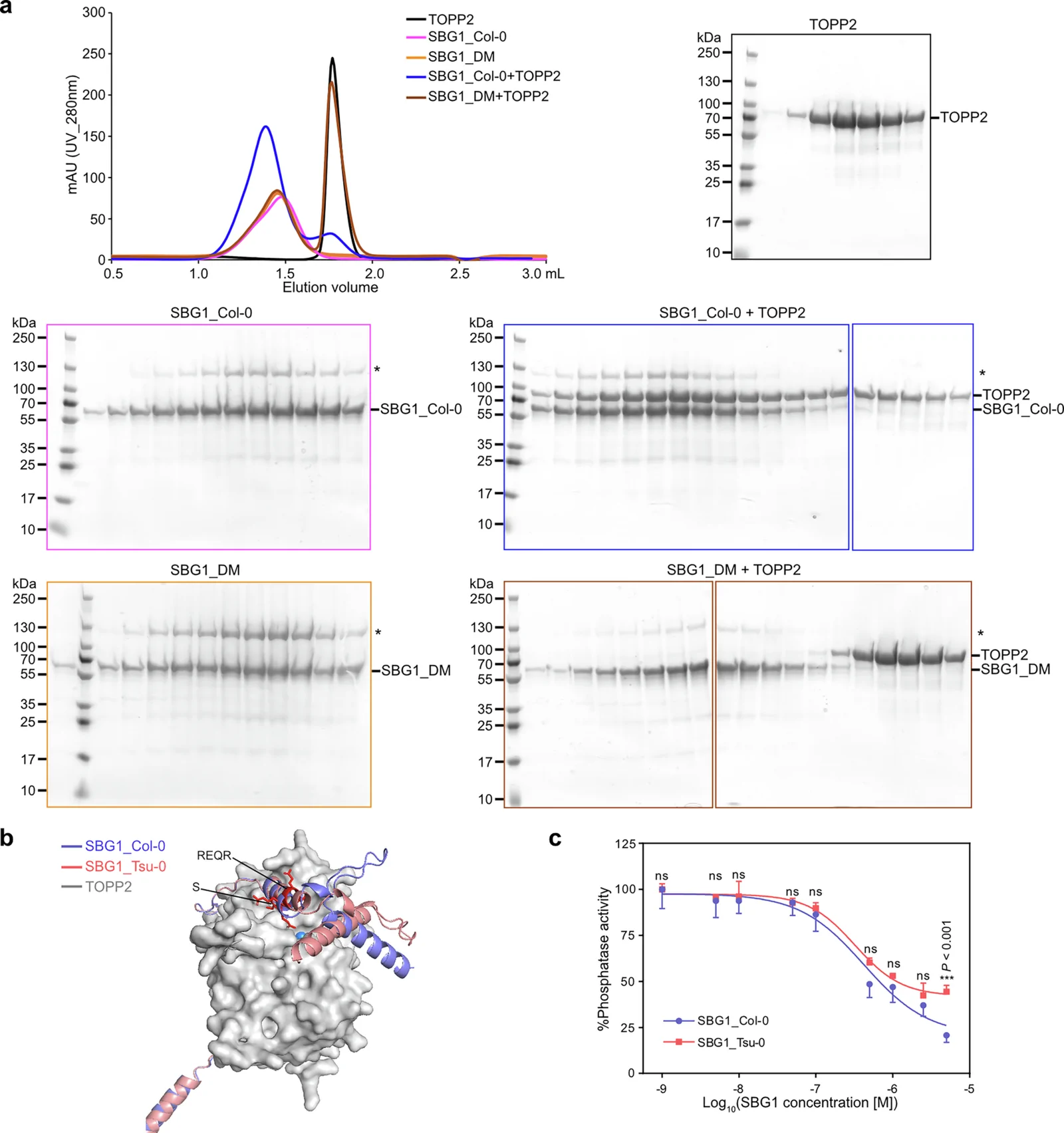
SBG1 binds to and inhibits TOPP2 *in vitro*. **a**, Analytic gel-filtration assay showing that only SBG1_Col-0 but not SBG1_DM binds to TOPP2. SDS-PAGEs showing the collected fractions of eluted peaks after gel filtration. Asterisks indicate the lager band which might be the dimer complex of SBG1 in all the samples. Experiment was repeated 2 times independently with similar results. **b**, Aligned AlphaFold predicted structure of SBG1 from Col-0 and Tsu-0 in complex with TOPP2. Side chains in the differential sites are shown in red. **c**, TOPP2 phosphatase activity assay showing comparable inhibition of TOPP2 by SBG1_Col-0 or SBG1_Tsu-0. Two-way ANOVA followed by Sidak’s test was used to assess the significance at each concentration (***P < 0.001, ns, not significant, *n* = 3 biological replicates). Individual numeric values and the exact *P*-values are provided in Source Data_Extended Data Fig. 9. Source data

TOPPs constitute a conserved family of serine/threonine phosphatases with broad functions in plants, including cell division, differentiation, salt tolerance and ABA signalling^29^. They are orthologues of Protein Phosphatase 1 (PP1) in animals and fungi, where their activity is modulated by regulatory subunits that interact through conserved RVxF and SILK motifs^30^. These regulatory interactions can either inhibit TOPPs activity by blocking substrate access (as seen for Inhibitor-2 (I2)) or recruit substrates while controlling access to the catalytic site (as observed for Kinetochore Null Protein 1 (KNL1))^29,31,32^. Strikingly, SBG1 and SBG1L1 both contain SILK and RVxF motifs, while SBG1L2 and SBG1L3 retain only the RVxF motif, suggesting that SBG1 proteins might act as TOPP regulators (Extended Data Fig. 7d). To explore this hypothesis, we first employed an in silico structural approach using AlphaFold3 (ref. ^33^), focusing on TOPP2, the most enriched interactor in our IP–MS dataset. The predicted complex showed high confidence with an interface predicted template modelling (ipTM) value of 0.85 and a predicted template modelling (pTM) of 0.79, comparable to the validated TOPP–I2 interaction^32,34^ (Fig. 4b and Extended Data Fig. 8e–g). These models predict that SBG1 interacts with TOPP2 via its central region containing both SILK and RVxF motifs (Fig. 4b and Extended Data Fig. 8e,f). Similar predictions were obtained for SBG1L1, whereas SBG1L2 and SBG1L3, which lack the SILK motif, show only partial overlap in their predicted binding interfaces (Extended Data Figs. 7d and 8g). In addition, the C termini of SBG1 homologues and the known TOPP inhibitor I2 are predicted to form similar structural covering over the TOPP2 catalytic site. This is particularly evident for SBG1 and SBG1L1, which possess extended C-terminal loops (Extended Data Fig. 8g), suggesting that SBG1 proteins may inhibit TOPP2 activity by sterically blocking access to its catalytic site. The differences in motif composition among SBG1 homologues and C termini provide a mechanistic rationale for their unequal genetic contributions. SBG1 and SBG1L1, the only family members containing both the SILK and RVxF motifs required for TOPP binding and extended C termini, contribute most strongly to the ABA-induced suberization response, whereas SBG1L2 and SBG1L3, lacking the SILK motif, have a weaker impact. This is reflected in the incremental phenotype of the quadruple mutant compared to the double mutant (Fig. 3i), supporting a model in which SBG1 family members regulate suberization in proportion to their predicted ability to interact with TOPPs. We next experimentally validated this interaction using WT SBG1 and mutant versions carrying substitutions in the RVxF motif (SBG1_SM, V63A/F65A) or in both SILK and RVxF motifs (SBG1_DM, C46A/L47A and V63A/F65A). Using AlphaFold, which is known to have a limited capacity to accurately predict the structural consequences of point mutations^35,36^, the predicted SBG1_SM-TOPP2 and SBG1_DM-TOPP2 complexes showed only a modest reduction in prediction confidence relative to the wild-type complex (Extended Data Fig. 8h). Despite these predictions, yeast two-hybrid assays confirmed that SBG1 interacts with both TOPP1 and TOPP2, whereas the SBG1_SM and SBG1_DM mutations weakened the interactions with TOPP1 and abolished those with TOPP2 (Fig. 4d,e). Consistently, analytic gel-filtration assays showed that wild-type SBG1 co-eluted with TOPP2, confirming a direct physical interaction, while the double mutant version SBG1_DM failed to bind TOPP2 (Extended Data Fig. 9a). To assess the functional relevance of this interaction, we performed in vitro phosphatase assays using recombinant proteins. Wild-type SBG1 inhibited TOPP2 activity in a concentration-dependent manner, whereas SBG1_DM had no effect, demonstrating that the interaction via the SILK and RVxF motifs is required for TOPP2 inhibition (Fig. 4f). We next examined whether allelic variation in *SBG1* coding sequence could modulate this inhibitory effect. Comparison of the *T* and *G* alleles by AlphaFold prediction revealed no detectable differences in the predicted SBG1–TOPP2 interaction (Extended Data Fig. 9b). Consistently, in vitro phosphatase assays using recombinant proteins from the two allelic variants showed similar TOPP2 inhibition (Extended Data Fig. 9c). These findings reinforce the hypothesis that functional differences between alleles arise from regulatory variation in the 3′UTR rather than from changes in the coding sequence. Together, these findings demonstrate that SBG1 physically interacts with TOPPs, particularly TOPP2, via conserved SILK and RVxF motifs, and acts as an inhibitory regulator of their phosphatase activity.

**Extended Data Fig. 10:**
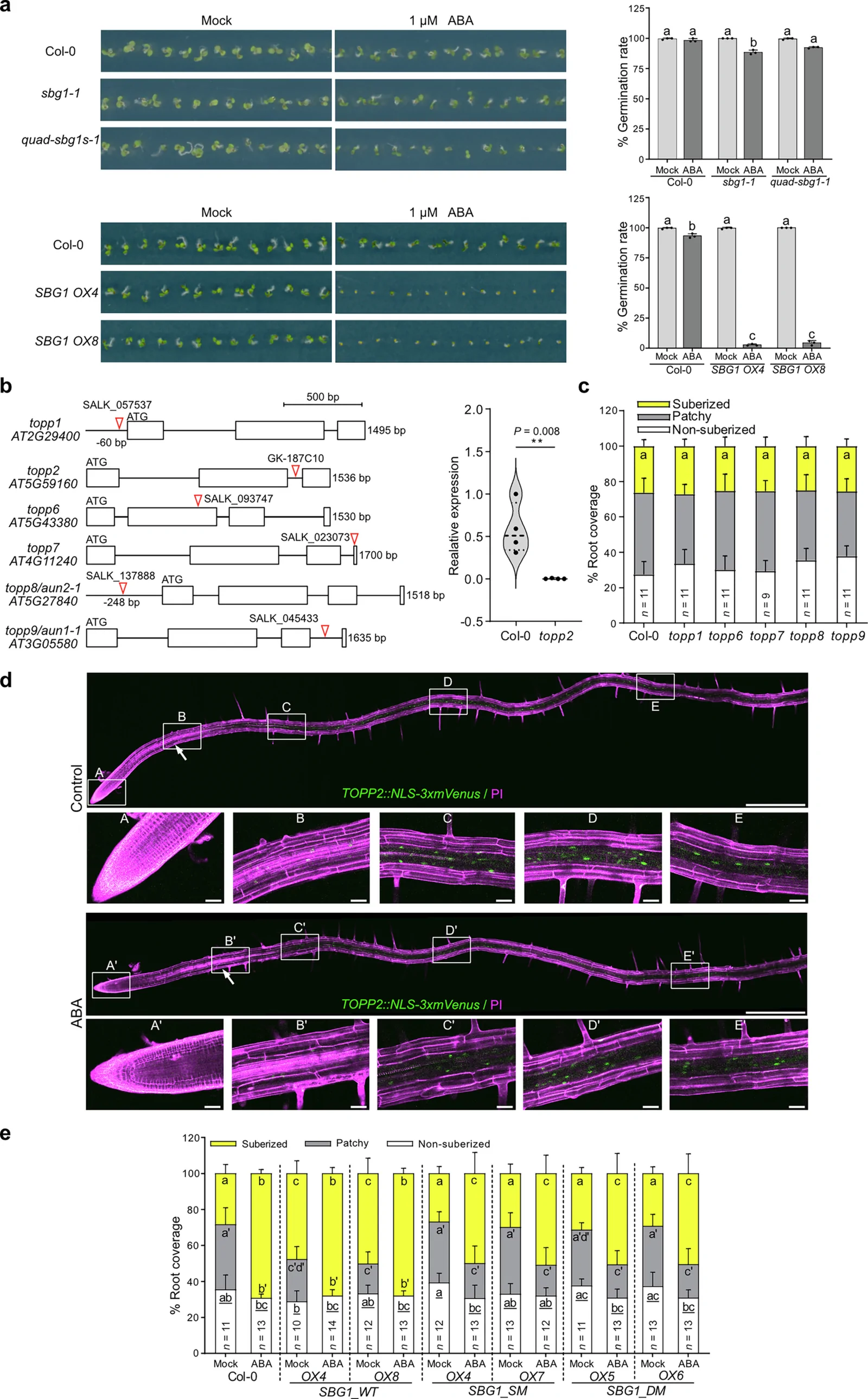
SBG1 promotes suberization through interaction with TOPPs. **a**, Seed germination assay. For each biological replicate 84 seeds per genotypes were sown on 1/2 MS or 1/2 MS supplemented with 1 µM ABA. Germination was scored after 96 hours. Data are shown as histograms with individual values overlaid, mean +/− SD, *n* = 3 biological replicates. One-way ANOVA followed by Tukey’s test was used to assess differences between genotypes and different letters indicate significant differences (*P* < 0.05). **b**, Schematic showing the position of the T-DNA insertions in *topp* mutants. Homozygous mutants were isolated by genotyping and T-DNA insertion sites were confirmed by sequencing (left panel). Violin plot showing the relative expression of *TOPP2* in Col-0 and *topp2* determined by qRT-PCR (right panel). Thick dashed lines in the violin plots indicate the median, while dotted lines represent the first and third quartiles. A two-tailed Student’s *t*-test was used to test the significance (**P < 0.01, *n* = 4 pool of approximately 60 roots). **c**, Suberin deposition pattern in *topp* mutants. Data are presented as stacked columns showing the mean percentage of each suberization zone +/− SD, with *n* shown on the graph. One-way ANOVA followed by a Tukey’s test was used for the suberized zone to compare genotype. No significant difference was detected (*P* > 0.05). **d**, Confocal images of *TOPP2::NLS-3xmVenus* lines treated with or without 1 µM ABA for 18 h. The full scan as well as higher resolution images are presented. mVenus signal is shown in green and PI in magenta. White arrows indicate the onset of mVenus signal. Scale bar, 500 µm. **e**, Suberin deposition pattern in transgenic lines overexpressing *SBG1_WT, SBG1_SM or SBG1_DM* under control (mock) or 1 µM ABA treatment for 16 h. 4-day-old seedlings were transferred to either 1/2 MS plates or 1/2 MS plate + ABA. Data are presented as in (**c**). Different letters indicate significant differences in the different suberized zones between genotypes and conditions with apostrophes and underlines for the patch and non-suberized zones respectively. **a-c,e**, Individual numeric values and the exact *P*-values are provided in Source Data_Extended Data Fig. 10. Source data

To our knowledge, TOPPs have not been previously implicated in suberin or polyester formation. However, previous studies showed that *topp* loss-of-function mutants are hypersensitive to ABA, and TOPP1 was reported to inactivate SNF1-related protein kinase 2.6 (SnRK2.6, also known as Open stomata (OST1)), a key positive regulator of ABA signalling^34,37^. Consistently, we observed that SBG1-mVenus overexpressing lines, but not *sbg1* mutants, displayed ABA hypersensitivity comparable to previously described *topp* mutants (Extended Data Fig. 10a)^37^. This suggests that SBG1 may inhibit TOPP activity, thereby modulating ABA signalling. We next examined suberin deposition in several *topp* mutants (*topp1*, *2, 6, 7, 8* and *9*) and found that only *topp2* displayed a marked increase in suberin deposition, with ectopic endodermal suberization near the root tip (Fig. 4g and Extended Data Fig. 10b,c). This phenotype closely resembled that of *UBQ10::SBG1-mVenus* overexpression lines and was opposite to the reduced suberization observed in *sbg1* mutants, supporting a model in which SBG1 promotes suberization by antagonizing TOPP2 function in ABA signalling. Consistent with this model, we found that *TOPP2* promoter is active in the endodermis, similarly to *SBG1*, but in contrast to *SBG1* is unresponsive to ABA (Fig. 4h and Extended Data Fig. 10d). This observation is further supported by our RNA-seq analysis, showing that *TOPP2* transcript levels in roots are unaffected by ABA treatment (Extended Data Fig. 7e). Together, these data suggest that TOPP2 is probably constitutively expressed in the endodermis. While we cannot exclude the possibility of ABA-dependent post-transcriptional regulation affecting TOPP2 protein abundance, our results support a model in which ABA-induced *SBG1* expression antagonizes constitutively expressed TOPP2 in the endodermal cells to promote suberin deposition. To determine the genetic relationship between the two genes, we performed epistasis analysis. The double mutant *sbg1topp2* displayed an ectopic suberization pattern indistinguishable from that of *topp2* single mutants (Fig. 4g), demonstrating that *TOPP2* is epistatic to *SBG1* and that SBG1 acts upstream of TOPP2 in this regulatory cascade. These findings support a model in which SBG1 inhibits TOPP2 activity, thereby alleviating its negative effect on ABA signalling and promoting suberization of endodermal cells. To further test this hypothesis, we generated plants overexpressing SBG1-mVenus mutated in either the RVxF motif (SBG1_SM) or in both SILK and RVxF motifs (SBG1_DM). In contrast to the WT construct, neither mutant induced suberin deposition (Fig. 4i). Moreover, both mutants showed reduced suberin induction in response to ABA, suggesting a partial dominant-negative effect (Extended Data Fig. 10e). Together with the in vitro analysis, these results support a model in which SBG1 promotes endodermal suberization through its interaction with TOPP2, probably by inhibiting its phosphatase activity. Our findings thus position SBG1 as a regulator of endodermal suberization acting via modulation of TOPP phosphatases.

## Discussion

Suberin deposition in roots has attracted growing attention due to its role as a protective barrier against drought and pathogens and as a potential contributor to underground carbon storage. In particular, suberization of the endodermis and exodermis forms a dynamic barrier for water and nutrients^2,8,25,38–42^. Key regulators of endodermal suberin deposition are conserved across vascular plants and were instrumental in the evolution of seed plants^5,40,43,44^. While substantial progress has been made in understanding the molecular pathway of suberin biosynthesis and regulation, most insights come from the *A. thaliana* reference accession Col-0. Moreover, no unbiased, forward genetic approach, aiming at identifying unknown players in suberization, had been performed. Previous studies on natural variation in suberin have focused on peridermal suberization in older roots^45^, linking chemical diversity in suberin to soil properties and moisture gradients, identifying 94 putative loci, with verification for *GPAT6*, which has a demonstrated role in suberin biosynthesis^27,45^. Here we targeted the genetic diversity underlying the highly plastic endodermal suberization to uncover mechanisms involved in nutrient acquisition and stress adaptation. Our screen revealed a strong association between endodermal suberization and accumulation of Rb, Mo, Cu and Zn in shoots grown on agar plates. Unexpectedly, K and Na, classical markers of endodermal suberin levels in Col-0, were not affected, emphasizing that Col-0 may not be representative of species-wide responses, particularly for traits linked to root developmental physiology. Similar accession-dependent divergences were previously reported for Zn and P deficiencies responses and for root growth response to hormones^46,47^. In this context, our data suggest that the previously established role of suberin as a protective barrier against Na cannot be generalized across accession. Instead, accession-specific regulatory differences may independently influence both endodermal suberization and growth responses under salt stress. In such a scenario, suberin would be neither intrinsically protective nor detrimental, but rather a correlated outcome of broader stress-response programmes that differ among accessions. While previous studies in Col-0 linked endodermal suberin to B, Rb, Cu and Mo accumulation^3,13,18^, a consistent impact on Zn was not shown before. Accessions with enhanced suberin accumulated less Zn, and several displayed increased sensitivity to Zn limitation, a phenotype that was either unchanged or alleviated when suberin was degraded. These findings align with earlier observations that Zn limitation downregulates suberization, a response proposed to facilitate Zn uptake^2^. Together, these results position endodermal suberin as a contributor to Zn homeostasis, with implications for nutrient adaptation strategies in plants, and highlight an accession-dependent trade-off between protective barrier function and nutrient acquisition.

Our GWAs analysis identified multiple loci associated with endodermal suberization, including two that surpassed the Bonferroni-corrected significance threshold and more than 20 additional loci exceeding the Benjamini–Hochberg threshold. Strikingly, none of the previously known regulators of suberin deposition were found among these loci, underscoring the strength of this unbiased approach in uncovering novel genetic determinants. Focusing on the strongest association on chromosome 1, we identified *SBG1*, a previously unannotated gene whose naturally occurring allelic variation in its 3′UTR impacts the pattern of suberin deposition across accessions, suggesting a major role in suberin-dependent environmental adaptation. Supporting a direct role in suberization, *SBG1* is expressed in the endodermis and is transcriptionally induced by ABA, a well-established hormonal regulator of endodermal suberin deposition, during both stress responses and root development^2,3,8,43^. Consistent with its expression pattern, *SBG1* regulates ABA-induced endodermal suberization and has a broad effect on the transcriptional response to ABA in roots, suggesting a regulatory role in the ABA response. We found that SBG1 physically interacts with the protein phosphatases TOPPs, particularly TOPP2, which is constitutively expressed in the endodermis and inhibits its activity in vitro. Since TOPPs are known negative regulators of ABA signalling^34,37^, our findings suggest that SBG1 interferes with TOPP activity, thereby locally relieving ABA repression and promoting suberin formation. The enhanced suberin phenotype observed in the *topp2* mutant, which phenocopies WT *SBG1* constitutive expression and is epistatic to *sbg1*, further supports TOPP2 as a central target of SBG1-mediated suberin regulation. Together, our results define a novel regulatory module that fine tunes ABA signalling in the endodermis to control suberin deposition. Given the evolutionary conservation of SBG1 across angiosperms (Supplementary Fig. 3a,b), this mechanism probably represents a fundamental and broadly relevant strategy for regulating root barrier properties in diverse plant species.

Together, our findings highlight the power of unbiased forward genetic approaches to uncover previously unknown regulators of endodermal suberin deposition. By revealing the genetic basis underlying natural variation in suberin deposition, we identify SBG1 as a central, previously uncharacterized regulator of this key trait. This work not only advances our understanding of the mechanisms governing root barrier formation but also provides a new conceptual and molecular framework to manipulate ABA response and suberin deposition for improving plant stress resilience.

## Methods

### Plant material

The 284 *Arabidopsis* accessions used in this study are listed in Supplementary Table 1 and represent a subset of the 1001 Genomes Project^48^, including 185 Salk accessions, 80 Max-Planck Institute accessions and the 19 MAGIC (Multiparent Advanced Generation Inter-Cross) founder lines^49^. The ER-GFP marker line was described previously^50^. T-DNA insertion lines *sbg1-1* (SALK_029983C), *frs6-2* (SALK_114017C)^51^, *at1g52540* (GK-090F10), *at1g52550* (SAIL_681_G01), *pldα2* (GK-212E06), *rbl5* (SALK_079189C), *at1g52590* (SAIL_894_H03.C), *at1g52600* (SALK_062346C), *topp1* (SALK_057537)^34,52^, *topp2* (GK-187C10)^37^, *topp6* (SALK_093747C)^52^, *topp7* (SALK_023073C)^52^, *topp8/aun2-1* (SALK_137888)^53^ and *topp9/aun1-1* (SALK_045433C)^52,53^ were obtained from the Nottingham *Arabidopsis* Stock Centre. Genotyping primers are listed in Supplementary Table 6. CRISPR-Cas9-generated mutants (*sbg1-2, sbg1-3, sbg1-4, sbg1;sbg1l1 and quad-sbg1*) were produced using guide RNAs described in the ‘Constructs and plant transformation’ section. Double mutants (*sbg1-1/topp2* and *sbg1-2/topp2*) were generated by crossing and identified by genotyping and sequencing. The following transgenic lines were generated in this study: *UBQ10::SBG1_acc* (*SBG1* from specified accessions), *UBQ10::SBG1-mVenus*, *UBQ10::SBG1_SM-mVenus, UBQ10::SBG1_DM-mVenus, SBG1::NLS-3xmVenus*, *SBG1::SBG1*, *SBG1::SBG1-mVenus, UBQ10::SBG1-mCherry*, *UBQ10::CYP86A1-mScarletI*/*UBQ10::SBG1-mVenus*, *UBQ10::TOPP2-mScarletI* /*UBQ10::SBG1-mVenus* and *TOPP2::NLS-3xmvenus*. Gene IDs are as follows: *SBG1*, *AT1G52565*; *SBG1L1*, *AT3G15760*; *SBG1L2*, *AT2G35850*; *SBG1L3*, *AT3G52360*; *TOPP1*, *AT2G29400*; *TOPP2*, *AT5G59160*; *TOPP6*, *AT5G43380*; *TOPP7*, *AT4G11240*; *TOPP8*, *AT5G27840*; *TOPP9*, *AT3G05580*; *GPAT5*, *AT3G11430*; *CYP86A1*, *AT5G58860*; *MYB41, AT4G28110*; *FRS6, AT1G52520*; *PLDα2, AT1G52570*; *RBL5, AT1G52580*; *CBF3*, *AT4G25480*; *ABF4*, *AT3G19290*; *LEA4-1*, *AT1G32560; I2, AT5G52200*.

### Constructs and plant transformation

CRISPR-Cas9 constructs targeting *SBG1* were designed using Benchling (https://www.benchling.com/) (Supplementary Table 6). Guide RNA oligonucleotides were annealed, BbsI (NEB, R3539S) digested and ligated into the pEN-Chimera vector^54^, and then recombined via Gateway Technology (Thermo Fisher) into a destination vector carrying *UBQ10::SpCas9* and a FAST Red selection marker^55^. Entry clones for expression constructs were generated by Gibson assembly following PCR amplification and cloned into a BamHI-digested (NEB, R3136S) pUC57-L4_EcoRV-XbaI-BamHI_R1 or into a NotI-digested (NEB, R0189S) pDONR221-*L1_SpeI-BglII-NotI-SphI-EcoRI_L2*. Newly generated entry clones include: *L4-proSBG1-R1* (3,000 bp promote*r*), *L4-proTOPP2-R1* (1,322 bp), *L1-SBG1-L2* (genomic, Col-0 no stop codon), *L1-SBG1_acc-L2* (genomic, from specified accession, with 1,500 bp 3′ region), *L1-TOPP2-L2* (genomic) and *L1-CYP86A1-L2* (CDS). Mutant versions *L1-SBG1_SM-L2* (V63A/F65A) and *L1-SBG1_DM-L2* (V63A/F65A, C46A/L47A) were synthesized by Genewiz and then recombined in the same way as for *UBQ10::SBG1-mVenus*. For yeast two-hybrid assays, CDS of *SBG1* 88-390 bp, *TOPP1* and *TOPP2* were cloned into pDONR221 via Gateway BP reactions then recombined into pGADT7 or pGBKT7 (TaKaRa). Mutant versions *(SBG1_SM* and *SBG1_DM -L2*) were synthesized by Genewiz and processed identically. For protein expression in *Escherichia coli* (*E. coli*), CDS of *SBG1* (Col-0 andTsu-0)*, SBG1_DM* and *TOPP2* were cloned by Gibson assembly into pETM41 (NovoPro) with an N-terminal 6×His-MBP tag. Previously described entry clones include *L4-pUBQ10-R1* (ref. ^56^) (promoter), *L1-nls-3xmVenus-L2* (ref. ^57^), *R2-mCherry-L3* (ref. ^57^), *R2-mVenus-L3* (ref. ^58^) and *R2-mScarletI-L3* (ref. ^59^) (CDS), and *R2-tHSP18.2-L3* (ref. ^60^) and *R2-tNOS-L3* (ref. ^61^) (terminators). Expression clones were assembled by Multisite Gateway recombination (Life Technologies) into pFR7m34GW (FAST Red) or pFG7m34GW (FAST Green)^60^. Primers are listed in Supplementary Table 6. Constructs were introduced into *Arabidopsis* via *Agrobacterium tumefaciens* GV3101 using the floral dip method^62^. At least 14 independent T1 lines were selected on the basis of FAST marker fluorescence^63^. Segregation, expression and phenotypes were analysed in T2 and experiments were performed on T3 homozygous lines with single insertions unless otherwise specified. All constructs generated in this study are available upon request to the corresponding author.

### Growth conditions

For in vitro experiments, seeds were surface sterilized with chlorine gas and sown on square plates. Unless stated otherwise, seedlings were grown on Murashige and Skoog medium (MS/2, Duchefa, M0254.0050, pH 5.7) containing 0.8% plant agar (Duchefa, P1001.1000) without sucrose. For nutrient deficiency assays, the medium was prepared using a 20× macronutrient stock solution (Sigma-Aldrich, M0654) and 2,000× micronutrient stocks to reach final concentrations of 9.4 mM KNO_3_, 10.3 mM NH_4_NO_3_, 625 mM KH_2_PO_4_, 1.49 mM CaCl_2_, 0.75 mM MgSO_4_, 50 µM H_3_BO_3_, 0.05 µM CuSO_4_·5 H_2_O, 50 µM MnSO_4_·H*2*O, 0.5 µM Na_2_MoO_4_·2 H*2*O, 15 µM ZnSO_4_·7 H_2_O, 2.5 µM KI, 0.05 µM CoCl_2_·6 H_2_O and 50 µM Fe-EDTA. 2-(*N*-Morpholino)ethanesulfonic acid (MES, 0.5 g l^−1^, Sigma-Aldrich, M3671) was added and the pH adjusted to 5.7 with KOH. For nutrient-deficient conditions, the corresponding element was omitted. For the suberin screening in 284 accessions, seeds were stratified for 7 days at 4 °C; for all other experiments, stratification was performed for 2–3 days. Seedlings were grown vertically at 22 °C under continuous light (~100 µE) and 40% relative humidity. For soil growth and salt treatment, plants were grown under long-day conditions (16 h light/8 h dark) at 20 ± 2 °C, 150–180 µE light intensity and 60% relative humidity. Unless otherwise specified, experiments were performed on 5-day-old seedlings.

### Suberin staining and pattern quantification

Suberin was visualized by whole-mount staining as previously described^3^. Seedlings were incubated in Fluorol Yellow 088 (0.01% w/v, lactic acid; Chem Cruz, sc-215052/Sigma-Aldrich, 27714) at 70 °C for 30 min in the dark, washed twice in water and counterstained with aniline blue (0.5% w/v; Sigma-Aldrich, 415049) for 30 min at r.t. in the dark, followed by 2 additional washes. Samples were mounted in 50% glycerol and imaged using a ZEISS Axio Zoom.V16 stereomicroscope with a GFP filter (excitation 450–490 nm, emission 500–550 nm). Whole-root images were acquired by tile scanning: a low-resolution scan (×7) was used to define the region of interest, followed by high-resolution acquisition (×63) with overlapping tiles (10%), which were stitched together. Suberin patterning was quantified by measuring the lengths of non-suberized, patchy and fully suberized zones along the root using Fiji^64^.

### Correlations of suberin pattern with climate factors and geographical origin

Climate data for the *Arabidopsis* accessions were obtained from AraCLIM (https://github.com/CLIMtools/AraCLIM)^65^ and extracted for the geographic origin of each accession. To account for population structure, genotyping data for 261 of the 284 accessions were retrieved from the 1001 Genomes project^48^ and imputed RegMap data^66^. Variant call format (VCF) files were merged using vcftools (v.1.19)^67^ and filtered for biallelic sites, minor allele frequency ≥0.05, and ≤10% missing data (vcftools v.0.1.16)^68^. The resulting genotype matrix was summarized by PCA using the SNPRelate v.1.42.0 R package^69^. The first two principal components (PC1, 17.45% variance; PC2, 7.8% variance) were included as covariates in linear models (lm) assessing associations between suberin traits and environmental variables: suberin^~^ environmental variable + PC1 + PC2. Models were fitted using both raw and scaled environmental variables to allow comparison of effect sizes. PC1 and PC2 were not significantly associated with suberin traits. Environmental variables were considered significant at *P* < 0.05 (two-sided *t*-test). Partial *R*^2^ values (Supplementary Table 2) were calculated as the difference between the full model (including environmental variables and PCs) and a model including only PC1 and PC2. All analyses were performed in R (v.4.5.0)^70^. The map displaying the geographical origin of the 284 accessions (Extended Data Fig. 2a) was generated with the ArcGIS Pro software (https://www.esri.com/en-us/arcgis/products/arcgis-pro/overview).

### PI staining and uptake assay

Propidium iodide (Sigma-Aldrich, P4170) was used as a cell wall counterstain and apoplastic tracer to assess Casparian strip functionality^24^. Seedlings were live-stained with 15 µM PI for 10 min in the dark and washed twice with water. Apoplastic barrier function was evaluated by counting the number of endodermal cells beyond the onset of elongation where PI uptake was blocked by Casparian strips. The onset of elongation was defined as the first endodermal cell with a length at least 3 times its width. Imaging was performed using a Leica DM6B epifluorescence microscope equipped with an I3 filter at ×20 magnification.

### Confocal microscopy and image analysis

Confocal imaging was performed using Leica SP8, Zeiss LSM 780 and Zeiss LSM 800 microscopes. Excitation and detection settings were: Leica SP8, GFP/mVenus (488 nm, 500–546 nm) and PI/mCherry (552 nm, 585–617 nm); Zeiss LSM 800, GFP/mVenus (488 nm, 500–546 nm) and PI (561 nm, 585–617 nm); Zeiss LSM 780, mVenus (514 nm, 515–561 nm) and PI (514 nm, 585–679 nm). Scanner and detector settings were kept constant across experiments. Images were analysed using ZEN2.3 software (ZEISS) and Fiji^64^ (http://fiji.sc/Fiji).

### Ionomic analysis

Elemental content in 7-day-old leaves (~100 per biological replicate, with 4 replicates per genotype) was measured by inductively coupled plasma mass spectrometry (ICP–MS). Dried samples were weighed, transferred to 15 ml metal-free tubes (VMR) and digested in 300 µl of 67% nitric acid (NORMATOM, VWR, 83872.29) containing 100 ppb indium (Inorganic Ventures, CGIN1) as an internal standard at 100 °C for 2 h. After cooling, samples were diluted to 15 ml with Milli-Q water (Merck Millipore). Elemental analysis was performed using an Agilent 7700X ICP–MS equipped with an autosampler in helium collision mode. Scandium (2 ppb, Inorganic Ventures, CGSC1) was added as an additional internal standard. Fifteen elements (B, Na, Mg, P, S, K, Ca, Mn, Fe, Co, Cu, Zn, Rb, Sr and Mo) were quantified. Calibration was performed using single-element standards (Inorganic Ventures, Suisse Technology Partners), and accuracy was validated using a certified reference material (NIST1515, apple leaves, Sigma-Aldrich). Element concentrations were calculated by external calibration, normalized to dry weight and processed using the instrument software.

### Chlorophyll quantification

Shoot material was collected, weighed and extracted in dimethylformamide (DMF; v:w = 10:1, Fisher, 10213810). Samples were centrifuged at 18,000 *g* for 1 min at r.t. and incubated at 4 °C overnight. After a second centrifugation (3 min, 18,000 *g*, r.t.), absorbances at 647 nm and 664 nm were measured using a Tecan Spark plate reader. Total chlorophyll content was calculated according to Porra’s method^71^.

### Genome-wide association analysis

GWAS was performed using the GWA-Portal (https://gwas.gmi.oeaw.ac.at/) on the fully suberized zone phenotype measured in 265 accessions (Supplementary Table 1). The imputed full sequence dataset (TAIR9), combining the 250,000 SNP array and the 1001 Genomes dataset, was used^72^. An accelerated mixed model (AMM) was applied, and significance was determined using a 10% threshold after Bonferroni–Hochberg correction. Candidate genes located within 15 kb of top SNPs with −log_10_*P* > 6 and minor allele count (mac) ≥ 15 are listed in Supplementary Table 1.

### *H*^2^ calculation

Broad-sense heritability was estimated from replicated phenotypic measurements across accessions (mean ~20 biological replicates per genotype, Source Data Fig. 1) using a variance partitioning approach. A linear mixed-effect model was fitted in R (lme4)^73^ with the formula: %Suberized ~ 1 + (1|Accession) + (1|Assay), where accession and experimental batch were treated as random effects. Variance components corresponding to genetic variance (*σ*^2^_*G*) and residual variance (*σ*^2^_*E*) were extracted and used to calculate *H*^2^ as: *H*^2 ^= *σ*^2^_*G*/(*σ*^2^_*G* + *σ*^2^_*E*) as previously described^74^.

### RNA secondary structure prediction

The mRNA secondary structure of the *SBG1* 3′UTR from different accessions were predicted using the RNAfold web server (http://rna.tbi.univie.ac.at/cgi-bin/RNAWebSuite/RNAfold.cgi). Predictions were performed with default settings, including minimum free energy (MFE) and partition function calculations, no isolated base pairs, dangling energies on both sides of helices and Turner energy parameters^75^.

### Suberin monomer analysis

Suberin monomers in roots were analysed by gas chromatography–mass spectrometry (GC–MS)^76^. Roots from 5-day-old seedlings (200–300 per replicate) were harvested and incubated in isopropanol containing 0.01% butylated hydroxytoluene (BHT, Sigma-Aldrich, B1378) at 85 °C for 10 min. Delipidization was performed sequentially using chloroform–methanol (2:1), chloroform–methanol (1:1) and methanol (all with 0.01% BHT), for 16 h and 8 h under agitation. Delipidized samples were then vacuum dried for 3 d. Depolymerization was carried out by base-catalysed *trans*-esterification in a 20-ml reaction mixture containing methyl acetate (3 ml), 25% sodium methoxide in dry methanol (5 ml) and dry methanol (12 ml). Methyl heptadecanoate (5 mg, Sigma-Aldrich, H4515) and *ω*-pentadecalactone (10 mg, Sigma-Aldrich, W2840009) were added as internal standards. Samples were incubated at 60 °C for 2 h, then extracted with dichloromethane (3.5 ml, Sigma-Aldrich, 650463), acidified with glacial acetic acid (0.7 ml) and washed with 0.9% NaCl in 100 mM Tris-HCl buffer, pH 8.0 (1 ml). Samples were vortexed for 20 s and centrifuged at 1,500 *g* for 2 min. The organic phase was collected, washed with 0.9% NaCl (2 ml), dried over sodium sulfate and concentrated under a nitrogen stream. Derivation was performed with *N*,*O*-bis(trimethylsilyl)trifluoroacetamide (BFTSA) in pyridine (1:1, v/v; Sigma-Aldrich, 15238 and 270970) at 70 °C for 1 h. Samples were analysed on an Agilent 6890N GC system coupled to a mass spectrometer and flame ionization detector using an HP-5MS column (J&W Scientific). The oven programme was: 50 °C for 2 min, ramp to 160 °C at 20 °C min^−1^, to 250 °C at 2 °C min^−1^, then to 310 °C at 10 °C min^−1^ (15 min hold). Unsubstituted C16 and C18 fatty acids were excluded from the analysis due to their widespread occurrence in plants and the environment.

### Protein sequence alignment and phylogenetic analysis

Protein sequences of SBG1, SBG1L1, SBGL2 and SBG1L3 were aligned using Clustal Omega in SnapGene (https://www.snapgene.com/). Orthologous SBG1 sequences were identified by BLAST search against the NCBI database and aligned using Clustal Omega (https://www.ebi.ac.uk/jdispatcher/msa/clustalo)^77^. Phylogenetic analysis was performed using ClustalW2 with neighbour-joining clustering and Phylip output format (https://www.ebi.ac.uk/jdispatcher/phylogeny/simple_phylogeny)^77^.

### RT–qPCR

Gene expression was analysed using roots from 7-day-old seedlings (~60 roots pooled per biological replicate; 3 replicates per genotype and condition). Samples were flash frozen in liquid nitrogen, ground using a tissue lyser, and total RNA was extracted with TRIzol (LifeTechnologies, 15596018) followed by purification with the RNeasy MinElute kit (Qiagen, 74204). Complementary (c)DNA synthesis was performed using the Maxima First Strand cDNA Synthesis kit (Thermo Fisher, K1671). qPCR was carried out on an Applied Biosystems QuantStudio5 system using SYBR Green master mix (Thermo Fisher, A25743). Expression levels were normalized to the housekeeping gene *Clathrin AP4M* (*AT4G24550*), and relative expression was calculated using the ${2}^{-\varDelta \varDelta {C}_{{\rm{t}}}}$ method^78^. Primer sequences are listed in Supplementary Table 6.

### RNA sequencing and analysis

Roots from 6-day-old seedlings (~250 roots pooled per biological replicate; 3 replicates per genotype and condition) were harvested and flash frozen in liquid nitrogen. RNA extraction and purification were performed as described for qPCR. Libraries were prepared and sequenced by Novogene using an Illumina platform (paired-end, 150 bp; ~6 Gb per sample). After quality controls, reads were mapped to the *Arabidopsis thaliana* TAIR10 genome using HISAT2 and gene-level count matrices were generated. Differential expression analysis was performed with DESeq2 using Benjamini–Hochberg correction (false discovery rate (FDR) < 0.05). Differentially expressed genes are listed in Supplementary Table 4, and raw data are available in the NCBI Sequence Read Archive (SRA) under accession PRJNA1438682. Gene Ontology enrichment analysis was conducted using g:Profiler (https://biit.cs.ut.ee/gprofiler/gost) on genes with adjusted *P* < 0.05.

### IP–MS analysis

Roots (~500) from 7-day-old seedlings expressing SBG1-mVenus or ER-GFP were harvested and ground for protein extraction in buffer (50 mM Tris-HCl pH 7.5, 150 mM NaCl, 2 mM EDTA, 5 mM dithiothreitol (DTT), 1 mM PMSF, 1% Triton X-100, protease inhibitor cocktail, Roche, 4693124001). GFP- or mVenus-tagged proteins were immunoprecipitated using GFP-Trap Magnetic Agarose beads (Chromotek, gmta-100). Beads were washed 3 times with wash buffer containing 50 mM Tris-HCl pH 7.5, 150 mM NaCl, 2 mM EDTA, 0.01% Triton X-100, and 3 times without EDTA and detergent, after which the remaining buffer was removed and the beads suspended in 100 µl 1 M urea in 50 mM ammonium bicarbonate. For proteomic analysis, proteins were reduced (10 mM DTT, 30 min, r.t.), alkylated (20 mM iodoacetamide, 30 min, dark, r.t.), quenched (with half the amount of DTT used for reduction, 10 min, dark, r.t.) and digested overnight at 25 °C with trypsin (300 ng, Promega) in 1 mM hydrochloric acid and 25 mM ammonium bicarbonate. Digestion was stopped with trifluoroacetic acid (0.5% final), samples centrifuged and a magnetic rack used to collect supernatants. Tryptic peptides were desalted using MonoSpin C18 columns (GL Sciences), eluted with 50% acetonitrile/0.1% trifluoroacetic acid, dried in a centrifugal vacuum concentrator and resuspended in 0.1% formic acid/2% acetonitrile.

LC–MS/MS analysis was performed on an Easy-nLC Orbitrap Fusion System (Thermo Fisher) with a nanospray source. Peptides were separated on a homemade C18 column (75 μm ID, 15–20 cm) using a 2 h gradient (3–30% solvent B, 300 nl min^−1^), followed with 2 rounds of washing in gradient (round 1: 90% solvent B, 1 min; incubation 5 min, 3% solvent B, 1 min; incubation 5 min; round 2: 90% solvent B, 1 min, incubation 2 min, 3% solvent B, 1 min, incubation 14 min). Solvent B was 0.1% (v/v) formic acid in 100% (v/v) acetonitrile. Data-independent acquisition (DIA) was performed with 1 full MS scan (resolution 120,000; automatic gain control (AGC) 50%; injection time (IT) 100 ms) and 41 overlapping windows covering 350–1,400 *m*/*z*. DIA segments were acquired at 30,000 resolution (AGC 200%; IT 54 ms maximum) with HCD fragmentation (normalized collision energy (NCE), 28%). Raw data were analysed using the directDIA workflow in Spectronaut (Biognosys) with default settings against the *Arabidopsis thaliana* Uniprot proteome (UP000006548).

### Co-immunoprecipitation and immunoblot analysis

For co-immunoprecipitation (Co-IP), 7-day-old seedlings (~200) co-expressing SBG1-mVenus with CYP86A1-mScarletI or TOPP2-mScarletI were harvested and ground in liquid nitrogen. Proteins were extracted in buffer (50 mM Tris-HCl pH 7.5, 150 mM NaCl, 2 mM EDTA, 5 mM dithiothreitol, 1 mM PMSF, 1% Triton X-100, protease inhibitor cocktail; Roche) and clarified by 4 centrifugations (16,000 *g*, 15 min, 4 °C). An aliquot (100 µl) was kept as input. Supernatant were incubated with GFP-Trap Magnetic Agarose beads for 2 h at 4 °C with rotation. Beads were washed 3 times with wash buffer containing 50 mM Tris-HCl pH 7.5, 150 mM NaCl, 2 mM EDTA and 0.01% Triton X-100, and 3 times without EDTA and detergent, and then eluted in 2× Laemmli buffer (100 µl buffer/50 µl beads) by boiling (95 °C, 5 min). Input (25 µl, boiled in 2× Laemmli, 95 °C, 5 min) and immunoprecipitated samples (2 µl) were separated by SDS–PAGE and transferred to PVDF membranes (GE Healthcare Lifesciences, 15269894). Immunoblots were probed with anti-GFP (1:4,000, JL-8, Clontech, 632381) or anti-RFP (1:4,000, Chromotek, 6G6) antibodies to detect mVenus and mScarletI, respectively, followed by HRP-conjugated anti-mouse secondary antibodies (1:10,000, Promega, W4021). Signals were detected using SuperSignal West Femto Substrate (Thermo Scientific, 34095) and visualized with a ChemiDoc imaging system (Amersham Imager 680).

### Alphafold3 analysis

Protein interactions between SBG1, SBG1L1, SBG1L2, SBG1L3, I2 and TOPP2 were predicted using AlphaFold3 model (https://alphafoldserver.com/). Full-length protein sequences, together with 2 Mn^2+^ ions were used as input. Predicted models were visualized and refined in PyMOL v.3.1.0 (https://www.pymol.org/).

### Yeast two-hybrid assay

Yeast two-hybrid (Y2H) assays were performed using Matchmaker Gold System (Clontech-TaKaRa) following manufacturer instructions. The Y2HGold strain was co-transformed with AD and BD constructs and selected on SD-LW (-Leu, -Trp) medium. For interaction assays, 5 µl of serial yeast dilutions were spotted on SD-LW and SD-LWH (-Leu, -Trp, -His) plates and incubated at 28 °C for 3 d.

### Protein expression and purification

Expression constructs for SBG1_Col-0, SBG1_Tsu-0, SBG1_DM and TOPP2 fused to an N-terminal 6×His-MBP tag were transformed into *E. coli* BL21 (DE3). Cultures (2 l Lysogeny Broth with kanamycin) were at 37 °C and cultured to optical density at 600 nm (OD_600_) of ~0.6, induced with 0.5 mM isopropyl *β*-D-1-thiogalactopyranoside (IPTG, MP Biomedicals, 114064102) and incubated overnight at 18 °C. Cells were collected by centrifugation and stored at −80 °C. Pellets were resuspended in lysis buffer (50 mM HEPES pH 8.0, 500 mM NaCl, 1 mM MnCl_2_, 2 mM DTT, 5% glycerol), lysed and clarified by centrifugation. The supernatant was filtered and applied to a 5 ml HiTrap MBP column equilibrated in lysis buffer. Proteins were eluted with buffer containing 20 mM maltose (without glycerol). Fractions containing target proteins were pooled, concentrated and further purified by size-exclusion chromatography (Superose 6 Increase 10/300 GL, GE Healthcare Life Sciences) in 25 mM HEPES pH 8.0, 500 mM NaCl, 1 mM MnCl_2_ and 2 mM DTT. Peak fractions were collected, flash frozen in liquid nitrogen and stored at −80 °C.

### Analytic gel filtration

Protein complex formation was assessed by mixing 100 µg of purified TOPP2 with 100 µg of wild-type or mutant SBG1 in buffer (25 mM HEPES pH 8.0, 500 mM NaCl, 1 mM MnCl_2_, 2 mM DTT). After incubation at 4 °C for 40 min, samples were subjected to size-exclusion chromatography on a Superose 6 3.2/300 column (GE Healthcare Life Sciences) equilibrated in the same buffer. Eluted fractions were analysed by SDS–PAGE followed by Coomassie staining (Bio-Rad, 161-0406).

### Phosphatase activity assay

TOPP2 phosphatase activity was measured as previously described^30^ using the phosphorylated peptide H3pT3 (Histone3 pT3: NH_2_-AR(pThr)KQTARKSTGGKAPRKQL-COOH, GenScript) as substrate. Free Pi release was quantified using Malachite Green reagent (MAK308, Sigma). Briefly, 25 µl of serially diluted SBG1 was mixed with 15 µl of TOPP2 (3.5 nM) or reaction buffer (background-signal control: 50 mM HEPES pH 8.0, 150 mM NaCl, 1 mM MnCl_2_ and 1 mM DTT) and incubated at r.t. for 30 min. The reaction was initiated by adding 10 µl of 500 µM H3pT3 peptide and incubated for 20 min at 30 °C. Reactions were stopped with 50 µl Malachite Green reagent and absorbance at 620 nm was measured after 30 min using a Tecan Spark plate reader. Relative activity was calculated by subtracting background signal and normalizing to TOPP2 alone. Half maximal inhibitory concentration (IC_50_) curves were generated by fitting data to a 4-parameter logistic model (Prism v.10.6.1).

### Statistical analysis

Statistical analysis and graph generation were performed using GraphPad Prism 10 (https://www.graphpad.com/), except for climate correlation analyses, which were conducted in R (v.4.5.0)^70^ as described above. All experiments were independently repeated at least 3 times with consistent results, except for proteomic analysis and Co-IP (2 independent experiments with 3 biological replicates) and RNA-seq (1 experiment with 3 independent pools of 250 roots). All *n* values correspond to biological replicates. Non-parametric Spearman correlations were performed in Prism using two-tailed tests with 95% confidence intervals. Cohen’s *d* was calculated as the difference between group means divided by the pooled standard deviation (s.d._pooled_): *d* = (*M*_2_−*M*_1_)/s.d._pooled_, where s.d._pooled_ = √(((*n*_1_−1) × s.d._1_^2^ +(*n*_2_−1) × s.d._2_^2^)/(*n*_1_ + *n*_2_−2)). Data were first tested for normality and homogeneity of variance, and appropriate parametric or non-parametric tests were applied accordingly, as specified in figure legends. All *P* values are provided in Source Data, with selected values indicated in the figures. PCA and clustering of ionomic data were performed using MVApp^79^ (https://mvapp.kaust.edu.sa/).

### Reporting summary

Further information on research design is available in the Nature Portfolio Reporting Summary linked to this article.

## Supplementary information

Supplementary Information Supplementary Figs. 1–3.

Reporting Summary

Supplementary Table 1 Suberin quantification of 284 *Arabidopsis* accessions.

Supplementary Table 2 Correlation of suberin and climate variables.

Supplementary Table 3 Ionomic analysis of extreme accessions.

Supplementary Table 4 RNA-seq of Col-0 and sbg1-1 with ABA treatment.

Supplementary Table 5 IP–MS analysis of SBG1-mVenus.

Supplementary Table 6 Primers used in this study.

Supplementary Data Numeric data and statistics for Supplementary Fig. 1.

## Source data

Source Data Fig. 1 Numeric data and statistics for Fig. 1.

Source Data Fig. 2 Numeric data and statistics for Fig. 2.

Source Data Fig. 3 Numeric data and statistics for Fig. 3.

Source Data Fig. 4 Numeric data and statistics for Fig. 4.

Source Data Extended Data Fig. 1 Numeric data and statistics for Extended Data Fig. 1.

Source Data Extended Data Fig. 2 Numeric data and statistics for Extended Data Fig. 2.

Source Data Extended Data Fig. 3 Numeric data and statistics for Extended Data Fig. 3.

Source Data Extended Data Fig. 4 Numeric data and statistics for Extended Data Fig. 4.

Source Data Extended Data Fig. 5 Numeric data and statistics for Extended Data Fig. 5.

Source Data Extended Data Fig. 6 Numeric data and statistics for Extended Data Fig. 6.

Source Data Extended Data Fig. 7 Numeric data and statistics for Extended Data Fig. 7.

Source Data Extended Data Fig. 9 Numeric data and statistics for Extended Data Fig. 9.

Source Data Extended Data Fig. 10 Numeric data and statistics for Extended Data Fig. 10.

## Data Availability

All data supporting the findings of this study are available in the main text and Supplementary Information. The raw RNAseq dataset is available in the NCBI Sequence Read Archive (SRA) under accession PRJNA1438682. Numerical values for all graphs and *P* values are provided as source data with this paper.
