# Supplementary material for: GWAS reveal SUBER GENE1-mediated suberization via type one phosphatases
Source: Nat Plants. 2026 May 5;12(5):1079–94. doi: 10.1038/s41477-026-02292-x (PMC13652734; doi:10.1038/s41477-026-02292-x)
Supplement: Supplementary file 1 [file 41477_2026_2292_MOESM1_ESM.pdf]

nature plants

Supplementary information

https://doi.org/10.1038/s41477-026-02292-x

GWAS reveal SUBER GENE1-mediated suberization via type one phosphatases

In the format provided by the authors and unedited

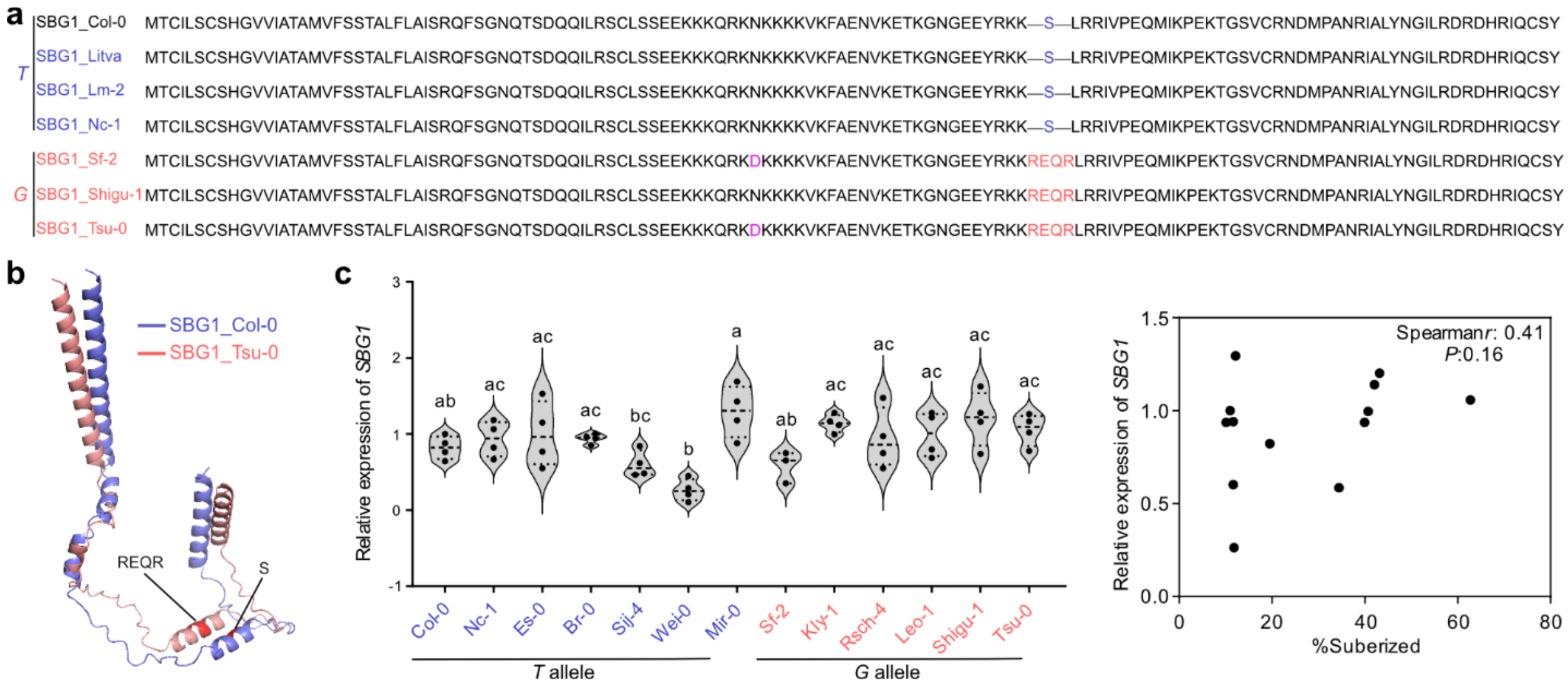

Supplementary Fig. 1 | Alignment of protein sequences of *SBG1* from *T* and *G* alleles. a, Protein sequence alignment of SBG1 in *T* and *G* alleles. The differential sites are marked in different colors. **b**, AlphaFold predicted structures of SBG1 from Col-0 and Tsu-0. The differential sites of "REQR" and "S" were marked in red. **c**, qRT-PCR analysis of *SBG1*'s expression in the accessions with *T* and *G* alleles. Data are shown as violin plot with individual values overlaid, $n = 4$. Thick dashed lines in the violin plots indicate the median, while dotted lines represent the first and third quartiles. One-way ANOVA followed by Tukey's test was used to assess the difference and different letters indicate significant differences between accessions ($P < 0.05$). Individual numeric values and the exact *P*-values for multiple comparisons are provided in Source Data_Extended Data_Figure 1_and_FigureS1. No significant correlation was shown between the expression level of *SBG1* and fully suberized pattern (two-tailed spearman correlation analysis, $P > 0.05$).

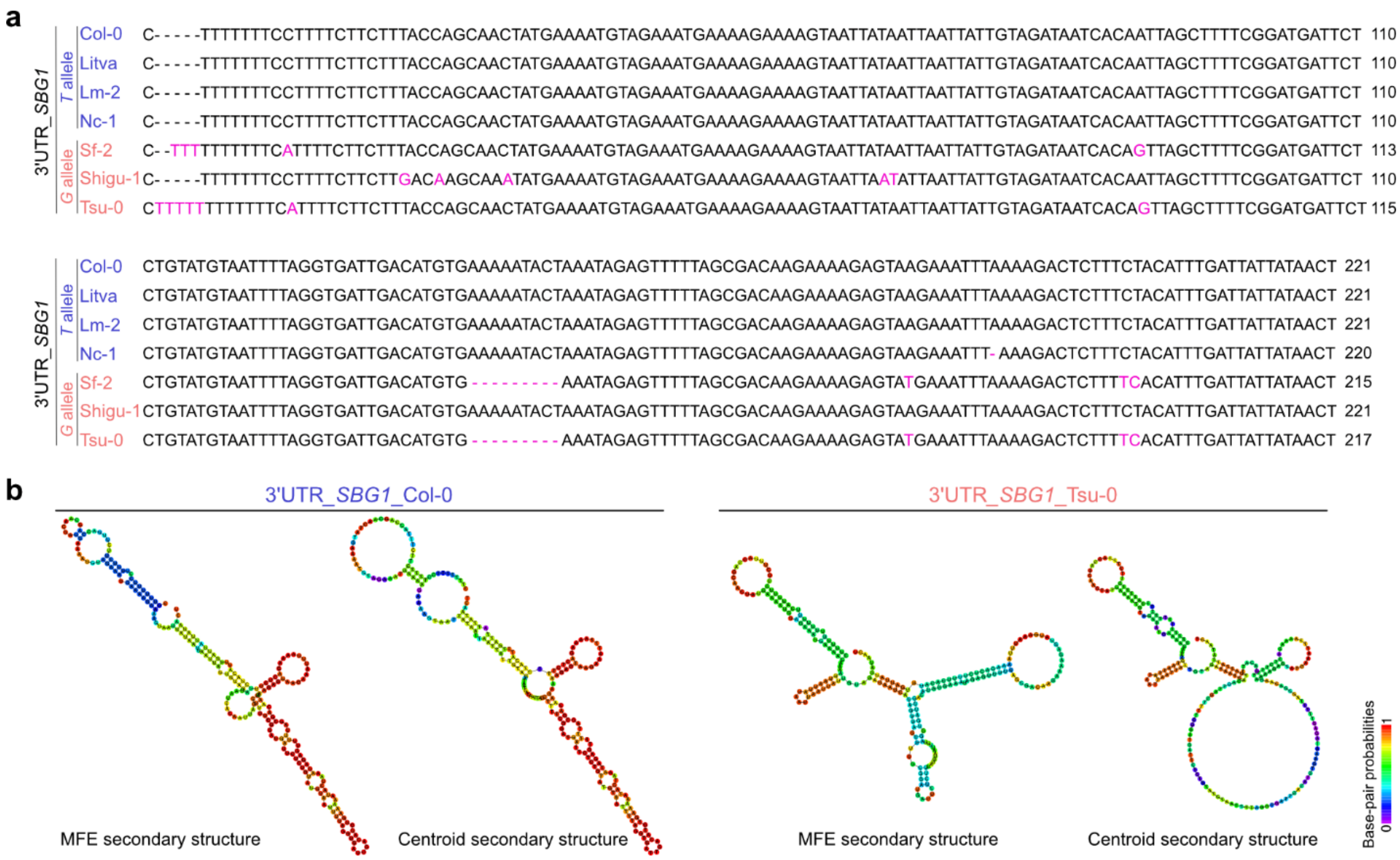

Supplementary Fig. 2 | Alignment of 3'UTR sequences of *SBG1* from *T* and *G* alleles. a, 3'UTR alignment of *SBG1* in *T* and *G* alleles. The differential sites are marked purple. **b**, RNAfold predicted RNA secondary structure of 3'UTRs of SBG1 from Col-0 (*T* allele) and Tsu-0 (*G* allele). Both MFE (minimum free energy) and centroid secondary structure are presented in color code of base-pair probabilities.

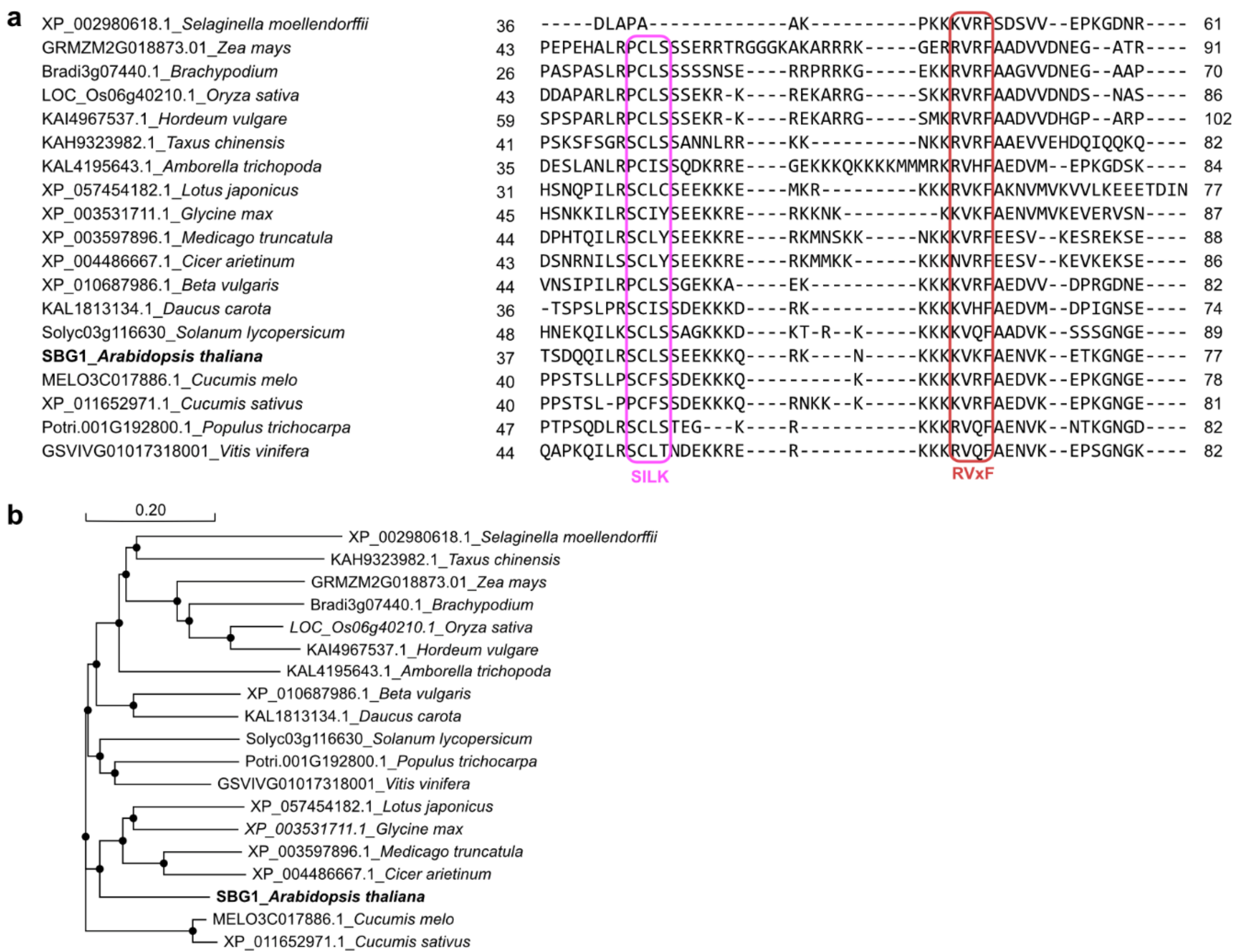

Supplementary Fig. 3 | Phylogenetic analysis of SBG1 and orthologs across species. a, Protein sequence alignment of SBG1 from *A. thaliana* and its orthologs from 18 plant species. The sequences of SBG1's orthologs were obtained by NCBI blast with highest protein similarity for each species. The SILK and RVxF motifs are highlighted in pink and red respectively. **b**, Phylogenetic analysis of SBG1 and its 18 orthologs. Scale bar indicates the branch lengths.
