# Supplementary material for: GWAS reveal SUBER GENE1-mediated suberization via type one phosphatases
Source: Nat Plants. 2026 May 5;12(5):1079–94. doi: 10.1038/s41477-026-02292-x (PMC13652734; doi:10.1038/s41477-026-02292-x)
Supplement: Supplementary file 2 [file 41477_2026_2292_MOESM2_ESM.pdf]

nature portfolio

Corresponding author(s): Marie Barberon

Last updated by author(s): Mar 26, 2026

Reporting Summary

Nature Portfolio wishes to improve the reproducibility of the work that we publish. This form provides structure for consistency and transparency in reporting. For further information on Nature Portfolio policies, see our Editorial Policies and the Editorial Policy Checklist.

Statistics

For all statistical analyses, confirm that the following items are present in the figure legend, table legend, main text, or Methods section.

n/a	Confirmed	
☐	☒	The exact sample size (n) for each experimental group/condition, given as a discrete number and unit of measurement
☐	☒	A statement on whether measurements were taken from distinct samples or whether the same sample was measured repeatedly
☐	☒	The statistical test(s) used AND whether they are one- or two-sided<br>*Only common tests should be described solely by name; describe more complex techniques in the Methods section.*
☐	☒	A description of all covariates tested
☐	☒	A description of any assumptions or corrections, such as tests of normality and adjustment for multiple comparisons
☐	☒	A full description of the statistical parameters including central tendency (e.g. means) or other basic estimates (e.g. regression coefficient) AND variation (e.g. standard deviation) or associated estimates of uncertainty (e.g. confidence intervals)
☐	☒	For null hypothesis testing, the test statistic (e.g. F, t, r) with confidence intervals, effect sizes, degrees of freedom and P value noted<br>*Give P values as exact values whenever suitable.*
☒	☐	For Bayesian analysis, information on the choice of priors and Markov chain Monte Carlo settings
☒	☐	For hierarchical and complex designs, identification of the appropriate level for tests and full reporting of outcomes
☐	☒	Estimates of effect sizes (e.g. Cohen's d, Pearson's r), indicating how they were calculated

Our web collection on statistics for biologists contains articles on many of the points above.

Software and code

Policy information about availability of computer code

Data collection	The SNP data used for GWAS were sequenced by the 1001 Genomes project and imputed on GWA-Portal website. The climate variables were from specified resources and integrated on AraCLIM website. Other data were generated in this study.
Data analysis	The GWAS was performed on GWA-Portal website. Suberin staining images were processed in Zeiss ZEN2.3 software and analyzed by Fiji. PCA and clustering analysis of ionomic data were performed on MVApp website. For IP-MS analysis, protein identification and quantification were analyzed using Spectronaut software (Biognosys). Confocal images were processed by Fiji. Guide RNAs for CRISPR-CAS9 constructs were designed using Benchling. mRNA secondary structures were predicted using the RNAfold web server. Protein structures were predicted by AlphaFold 3 and modified with PyMOL V3.1.0. SnapGene and ClustalW2 website was used for DNA and protein sequence analysis and alignment. GO analysis was conducted with g:Profiler. Sequenced were align using Clustal Omega in SnapGene and Phylogenetic analysis was performed using ClustalW2 ClustalW2 with neighbour-joining clustering and Phylip output format. All website are mentioned in the method section. All other data analysis were performed with GraphPad Prism 10.

For manuscripts utilizing custom algorithms or software that are central to the research but not yet described in published literature, software must be made available to editors and reviewers. We strongly encourage code deposition in a community repository (e.g. GitHub). See the Nature Portfolio guidelines for submitting code & software for further information.

Data

Policy information about availability of data

All manuscripts must include a data availability statement. This statement should provide the following information, where applicable:

- Accession codes, unique identifiers, or web links for publicly available datasets
- A description of any restrictions on data availability
- For clinical datasets or third party data, please ensure that the statement adheres to our policy

All data supporting the findings of this study are available in the main text and Supplementary Information. The raw RNAseq dataset is available in the NCBI Sequence Read Archive (SRA) under accession PRJNA1438682. Numerical values for all graphs and P-values are provided as Source Data. All genes and proteins mentioned in this study can be found in TAIR (https://www.arabidopsis.org/) or NCBI database (https://www.ncbi.nlm.nih.gov/). The corresponding gene IDs are: SBG1, AT1G52565; SBG1L1, AT3G15760; SBG1L2, AT2G35850; SBG1L3, AT3G52360; TOPP1, AT2G29400; TOPP2, AT5G59160; TOPP6, AT5G43380; TOPP7, AT4G11240; TOPP8, AT5G27840; TOPP9, AT3G05580; GPAT5, AT3G11430; CYP86A1, AT5G58860; MYB41, AT4G28110; FRS6, AT1G52520; PLDα2, AT1G52570; RBL5, AT1G52580; CBF3, AT4G25480; ABF4, AT3G19290; LEA4-1, AT1G32560; I2, AT5G52200.

Research involving human participants, their data, or biological material

Policy information about studies with human participants or human data. See also policy information about sex, gender (identity/presentation), and sexual orientation and race, ethnicity and racism.

Reporting on sex and gender	*Use the terms sex (biological attribute) and gender (shaped by social and cultural circumstances) carefully in order to avoid confusing both terms. Indicate if findings apply to only one sex or gender; describe whether sex and gender were considered in study design; whether sex and/or gender was determined based on self-reporting or assigned and methods used. Provide in the source data disaggregated sex and gender data, where this information has been collected, and if consent has been obtained for sharing of individual-level data; provide overall numbers in this Reporting Summary. Please state if this information has not been collected. Report sex- and gender-based analyses where performed, justify reasons for lack of sex- and gender-based analysis.*
Reporting on race, ethnicity, or other socially relevant groupings	*Please specify the socially constructed or socially relevant categorization variable(s) used in your manuscript and explain why they were used. Please note that such variables should not be used as proxies for other socially constructed/relevant variables (for example, race or ethnicity should not be used as a proxy for socioeconomic status). Provide clear definitions of the relevant terms used, how they were provided (by the participants/respondents, the researchers, or third parties), and the method(s) used to classify people into the different categories (e.g. self-report, census or administrative data, social media data, etc.) Please provide details about how you controlled for confounding variables in your analyses.*
Population characteristics	*Describe the covariate-relevant population characteristics of the human research participants (e.g. age, genotypic information, past and current diagnosis and treatment categories). If you filled out the behavioural & social sciences study design questions and have nothing to add here, write "See above."*
Recruitment	*Describe how participants were recruited. Outline any potential self-selection bias or other biases that may be present and how these are likely to impact results.*
Ethics oversight	*Identify the organization(s) that approved the study protocol.*

Note that full information on the approval of the study protocol must also be provided in the manuscript.

Field-specific reporting

Please select the one below that is the best fit for your research. If you are not sure, read the appropriate sections before making your selection.

☒ Life sciences ☐ Behavioural & social sciences ☐ Ecological, evolutionary & environmental sciences

For a reference copy of the document with all sections, see nature.com/documents/nr-reporting-summary-flat.pdf

Life sciences study design

All studies must disclose on these points even when the disclosure is negative.

Sample size	Sample sizes were chosen following common practice in the field. Sample size for experiments are indicated in figure legends or directly on the graphs and in the text. No statistical method was used to predetermine sample size. Generally sample size was chosen empirically, based on prior experience on how big a sample size must be to most probably obtain a reproducible, statistically significant result. For ionomic and suberin monomer analysis, n=4, each sample corresponding to a pool of approximately 100 and 250 seedlings respectively. For qRT-PCR, RNAseq and germination assay, n=3, each sample corresponding to a pool of approximately 60 and 250 individuals respectively.
Data exclusions	No data was excluded from the analysis.
Replication	All experiments were conducted independently at least 3 times, yielding consistent results, except for the proteomic and Co-IP analyses

Replication	where 2 independent experiments with 3 biological replicates were analyzed and the RNAseq performed in one experiment with 3 biological replicates of approximately 250 roots each
Randomization	The plates for growing Arabidopsis were randomized and their positions in growth chambers were also shifted randomly every other day during the growing phase. For transgenic lines and CRISPR mutants several independent lines were analyzed to minimize bias and off-target effects. Samples were allocated based on predefined genotypes and/or treatment conditions (e.g., wild type versus mutant lines, or control versus treatment), and therefore randomization was not applicable. All genotypes were grown and analyzed in parallel under identical experimental conditions to minimize potential confounding effects. Environmental variables (e.g., growth conditions, plate position, and handling) were kept consistent across groups, and where applicable, experiments included multiple biological replicates and independent repeats to ensure robustness.
Blinding	All suberin analysis after staining which needed manual analysis to quantify suberin pattern were designed in blind with numbers assigned to samples. All other experiments were not subjected to blinding following common practice in the field.

Reporting for specific materials, systems and methods

We require information from authors about some types of materials, experimental systems and methods used in many studies. Here, indicate whether each material, system or method listed is relevant to your study. If you are not sure if a list item applies to your research, read the appropriate section before selecting a response.

Materials & experimental systems

n/a	Involved in the study	
☐	☒	Antibodies
☐	☒	Eukaryotic cell lines
☒	☐	Palaeontology and archaeology
☒	☐	Animals and other organisms
☒	☐	Clinical data
☒	☐	Dual use research of concern
☐	☒	Plants

Methods

n/a	Involved in the study	
☒	☐	ChIP-seq
☒	☐	Flow cytometry
☒	☐	MRI-based neuroimaging

Antibodies

Antibodies used	Commercially available antibodies used in this study: anti-GFP (Clontech, JL-8, CAT#632381), anti-RFP (Chromotek, CAT#6G6) and anti-mouse IgG (H+L) HRP-conjugated secondary antibodies (Promega, CAT# W4021)
Validation	AntiGFP (JL-8): https://www.takarabio.com/documents/Certificate%20of%20Analysis/632380/632380-632381-070313.pdf?srsltid=AfmBOorhfiyw9HnBLRAxhFSHyWgbXrGHCLudOS8wfHayyL-5SAviF18i<br>Anti-RFP (6G6): https://www.ptglab.com/products/RFP-antibody-6G6.htm?srsltid=AfmBOopofxofdDynvnEOAjkS7EVBza6fwNphCnbziAMxCCspQGMzkGim<br>Anti-mouse gG (H+L) HRP-conjugated secondary antibodies (W4021): https://ch.promega.com/products/protein-detection/primary-and-secondary-antibodies/anti_mouse-igg-h-and-l-hrp-conjugate/?catNum=W4021

Eukaryotic cell lines

Policy information about cell lines and Sex and Gender in Research

Cell line source(s)	For yeast two-hybrid assay, the yeast strain (Y2HGold) from Matchmaker Gold Yeast Two-Hybrid System (Clontech; TaKaRa, Tokyo, Japan) was used.
Authentication	The yeast strain being commonly used for yeast two-hybrid analysis,was therefore not authenticated in this study.
Mycoplasma contamination	The yeast strain was not tested for mycoplasma contamination.
Commonly misidentified lines (See ICLAC register)	No commonly misidentified cell line was used in this study.

Plants

Seed stocks

All the Arabidopsis accessions and insertion lines were acquired from Nottingham Arabidopsis Stock Centre and their stock numbers as well as locations are indicated in the supplementary table or in the method section.

Novel plant genotypes

For transgenic lines, constructs were introduced into Arabidopsis using Agrobacterium tumefaciens strain GV3101 and the floral dip method. For each construct, at least 14 T1 seeds were selected based on fluorescence (FAST Red or FAST Green). Segregation ratios, gene expression levels, and phenotypes were analyzed across 14 independent T2 lines. All experiments were conducted on T3 homozygous lines with mono-insertional events unless otherwise noted.
For Crispr-Cas9 edited mutants, egg cell-specific promoter was used to drive SpCas9 with intron. The Cas9 was isolated in T2 by segregation. All the T3 Crispr lines without Cas9 were genotyped and confirmed. The sequences for guide RNAs are shown in the supplementary table. The edited sequences are shown in the extended figure.

Authentication

All the transgenic lines and mutants were confirmed by genotyping. Transgenic line with fluorescent tags were also examined by microscopy analysis. For the T-DNA and Crispr mutants, the insertion and editing sites were firstly confirmed by genotyping, then the expression level was also analyzed by qRT-PCR. For the T-DNA or Crispr mutants, at least two alleles showing similar phenotype were analyzed to exclude the multi-insertion or off-target effect. In addition, complementation lines were generated for the key mutant.
